# Gene‐lifestyle interaction on risk of type 2 diabetes: A systematic review

**DOI:** 10.1111/obr.12921

**Published:** 2019-09-02

**Authors:** Stefan Dietrich, Simone Jacobs, Ju‐Sheng Zheng, Karina Meidtner, Lukas Schwingshackl, Matthias B. Schulze

**Affiliations:** ^1^ Department of Molecular Epidemiology German Institute of Human Nutrition Potsdam Rehbruecke Nuthetal Germany; ^2^ MRC Epidemiology Unit School of Clinical Medicine University of Cambridge Cambridge UK; ^3^ School of Life Sciences Westlake University Hangzhou China; ^4^ German Center for Diabetes Research (DZD) München‐Neuherberg Germany; ^5^ Institute for Evidence in Medicine Faculty of Medicine and Medical Center—University of Freiburg Freiburg Germany; ^6^ University of Potsdam Institute of Nutritional Sciences Nuthetal Germany

**Keywords:** diet, gene‐lifestyle interaction, incident type 2 diabetes, physical activity, weight loss intervention

## Abstract

The pathophysiological influence of gene‐lifestyle interactions on the risk to develop type 2 diabetes (T2D) is currently under intensive research. This systematic review summarizes the evidence for gene‐lifestyle interactions regarding T2D incidence. MEDLINE, EMBASE, and Web of Science were systematically searched until 31 January 2019 to identify publication with (a) prospective study design; (b) T2D incidence; (c) gene‐diet, gene‐physical activity, and gene‐weight loss intervention interaction; and (d) population who are healthy or prediabetic. Of 66 eligible publications, 28 reported significant interactions. A variety of different genetic variants and dietary factors were studied. Variants at *TCF7L2* were most frequently investigated and showed interactions with fiber and whole grain on T2D incidence. Further gene‐diet interactions were reported for, eg, a western dietary pattern with a T2D‐GRS, fat and carbohydrate with *IRS1* rs2943641, and heme iron with variants of *HFE*. Physical activity showed interaction with *HNF1B*, *IRS1*, *PPARγ*, *ADRA2B*, *SLC2A2*, and *ABCC8* variants and weight loss interventions with *ENPP1*, *PPARγ*, *ADIPOR2*, *ADRA2B*, *TNFα*, and *LIPC* variants. However, most findings represent single study findings obtained in European ethnicities. Although some interactions have been reported, their conclusiveness is still low, as most findings were not yet replicated across multiple study populations.

**Abbreviations**
DESIR: Epidemiological Study on the Insulin Resistance Syndrome
DPP: Diabetes Prevention Project
DPS: Diabetes Prevention Study
EPIC: European Prospective Investigation into Cancer and Nutrition
G × D: gene‐diet
G × E: gene‐environment
G × L: gene‐weight loss due to lifestyle changes
G × PA: gene‐physical activity
GRS: gene risk score
HPFS: Health Professionals Follow‐Up Study
KOGES: Korean Genome and Epidemiology Study
LTPA: leisure‐time physical activity
KARE: KOGES and the Korea Association Resource
MDCS: Malmö Diet and Cancer study
NOS: Newcastle‐Ottawa Quality Assessment Scale
NHS: Nurses' Health Study
PA: physical activity
PREDIMED: Prevención con Dieta Mediterránea
PRISMA: Preferred Reporting Items for Systematic Reviews and Meta‐Analyses
RCT: randomized controlled trial
SDPP: Stockholm Diabetes Prevention Program
T2D: type 2 diabetes mellitus

## 1 INTRODUCTION

Type 2 diabetes mellitus (T2D) represents an important health problem, causing enormous costs and individual burden, in part due to related macrovascular and microvascular complications.^1^ A fundamental understanding of the complex pathogenesis of T2D is essential to enable earliest diagnosis and improved therapies and preventive measures. The development of T2D is closely linked with unhealthy lifestyle. Of the lifestyle factors that define the personal way of living, overweight is the major risk factor for T2D.^1^, ^2^ Moreover, diet quality^3^ and physical activity (PA)^4^ are important lifestyle determinants of T2D risk independent of their effect on body weight. Susceptibility to these three modifiable lifestyle exposures, however, depends on genetic factors,^5^ which may interact with the lifestyle exposures. So‐called gene‐environment (G × E) interactions are in an epidemiological context defined as a combined risk effect of two exposures (genetic and environmental) on the outcome which is higher or less than the sum or product of the individual exposure effects.^6^ In a biological context, G × E interactions are defined as the coparticipation of two exposures in the same causal mechanism to outcome development.^7^ Identifying T2D risk subgroups based on genetic characteristics, which are especially sensitive to specific foods or nutrients, PA, or weight loss, may help to develop more individualized and targeted intervention strategies.

The evidence of G × E interaction in relation to T2D has been highlighted by several recent reviews.^8^, ^9^, ^10^, ^11^, ^12^, ^13^ However, to our knowledge, no systematic review has yet been published, which has extensively studied G × E interactions of lifestyle exposures regarding the risk to develop T2D. In 2007, Frank et al^8^ published a systematic review about G × E interactions on several outcomes, but at that time only a few of the identified studies included T2D incidence as outcome. A second systematic review was published in 2017 by Li et al,^14^ however, with the focus on gene‐macronutrient interaction only. In addition, in many other nonsystematic reviews,^9^, ^10^, ^11^, ^12^, ^13^ only part of the previous weight of evidence was based on prospective studies resulting in limited evidence in terms of temporal sequence of G × E interactions on the risk to develop T2D.

Several new findings of G × E interactions on the risk to develop T2D were recently reported by prospective studies which as well have not yet been systematically summarized. Hence, we aimed to systematically review the current state of evidence relating to G × E interaction and T2D incidence. We focused on prospective studies investigating interactions of genetic variants with diet (G × D), PA (G × PA), or weight loss due to lifestyle changes (G × L).

## 2 METHODS

This review was registered in PROSPERO (www.crd.york.ac.uk/prospero/index.asp, identifier CRD42015023898) and followed the Preferred Reporting Items for Systematic Reviews and Meta‐Analyses (PRISMA) guidelines 15.

### 2.1 Search strategy

Literature published through 31 January 2019 was systematically searched by three authors (S.J., J.S.Z., and S.D.) using the electronic databases PubMed, EMBASE, and ISI Web of Science, with restriction to English written publications. MeSH terms and other terms were used (list S1). Moreover, the reference lists of reviewed articles were checked to identify further eligible publications. S.J., J.S.Z., and S.D. screened titles, abstracts, and full texts in parallel, with disagreement resolved by consensus.

### 2.2 Study selection

Studies were included if they met the following criteria: (a) prospective study design (cohort studies and randomized controlled trials [RCTs]), (b) outcome: T2D incidence, (c) populations who are healthy or prediabetic, and (d) a description of G × D, G × PA, or G × L interaction. Non‐English literature and studies on populations with specific diseases (eg, cancer, cardiovascular diseases) and/or medications (eg, anticancer) were excluded. Moreover, we excluded studies without the application of a formal statistical test for interaction (Table S4). Studies with significant as well as not significant interaction terms were treated equally. Unpublished material was not considered.

### 2.3 Data extraction

Data extraction was conducted by S.J. or S.D. for the following information: title, authors, publication year, study name, study design, number of participants, ethnicities, age, gender, country, follow‐up time, exposures, exposure assessment methods, interaction terms, and *P* values and risk estimators of interactions.

### 2.4 Reporting strategy

Significant and nonsignificant interaction findings were objectively equally treated in this review.

### 2.5 Risk of bias and quality assessment

The risk of bias for cohort study publications was assessed by the Newcastle‐Ottawa Quality Assessment Scale (NOS).^16^ Thereby, three parameters of quality were investigated: selection, comparability, and outcome assessment including eight subitems that result in a maximum judgment score of 9. Studies were classified as low quality (0 to 3 points), moderate quality (4 to 6 points), and high quality (7 to 9 points).

The Cochrane Collaboration's tool was applied to assess risk of bias for RCTs.^17^ Assessed were: random sequence generation, allocation concealment, performance and detection bias, attrition bias, reporting bias, and funding bias. The risk of bias was judged either as low (with at least three items at low risk and one item at high risk of bias), high (with at least two items at high risk), or moderate/unclear (all other ratings).

The specific methodological quality of G × E interaction research was assessed by a score following quality criteria important for genetic association studies.^18^ This score requested eight items (Table S1): interaction as primary study goal, test for interaction, correction for multiple testing, correction for ethnicity, Hardy‐Weinberg equilibrium, test for group similarity at baseline, sample size, and sufficient details of study procedure. Points ranging from −8 to 8 were given to rate quality as follows: high quality (6 to 8 points), intermediate quality (2 to 5 points), and poor quality (−8 to 1 points).

## 3 RESULTS

**Figure 1:**
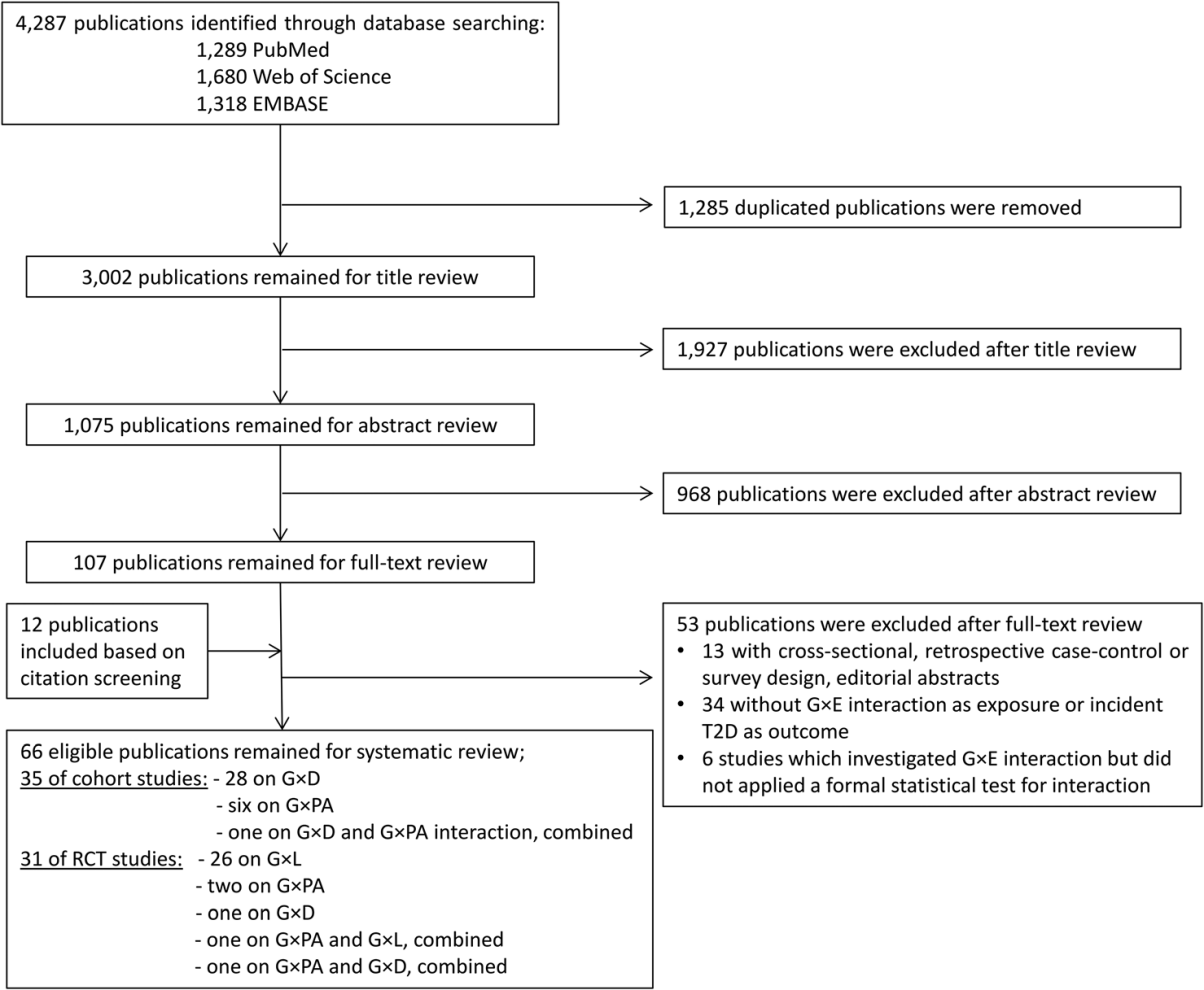
Flow chart of study selection. Abbreviation: D, diet; E, environment; G, gene; L, weight reduction due to lifestyle; PA, physical activity; RCT; randomized control trial; T2D, type 2 diabetes mellitus

Out of 3002 screened publications (Figure 1), 1075 and 107 publications were assessed during abstract and full text screening. Inter‐rater agreement was κ = 0.72 and κ = 0.76 for title and abstract screening, respectively. Overall, 66 eligible publications were identified including 35 publication from cohort studies^14^, ^19^, ^20^, ^21^, ^22^, ^23^, ^24^, ^25^, ^26^, ^27^, ^28^, ^29^, ^30^, ^31^, ^32^, ^33^, ^34^, ^35^, ^36^, ^37^, ^38^, ^39^, ^40^, ^41^, ^42^, ^43^, ^44^, ^45^, ^46^, ^47^, ^48^, ^49^, ^50^, ^51^, ^52^ and 31 from RCTs.^53^, ^54^, ^55^, ^56^, ^57^, ^58^, ^59^, ^60^, ^61^, ^62^, ^63^, ^64^, ^65^, ^66^, ^67^, ^68^, ^69^, ^70^, ^71^, ^72^, ^73^, ^74^, ^75^, ^76^, ^77^, ^78^, ^79^, ^80^, ^81^, ^82^, ^83^ Among the publications from cohort studies, 28 investigated G × D,^14^, ^19^, ^20^, ^21^, ^22^, ^23^, ^24^, ^25^, ^26^, ^27^, ^28^, ^29^, ^30^, ^31^, ^32^, ^33^, ^34^, ^35^, ^36^, ^37^, ^38^, ^39^, ^40^, ^41^, ^42^, ^43^, ^44^, ^45^ six G × PA,^47^, ^48^, ^49^, ^50^, ^51^, ^52^ and one combined G × D and G × PA interactions.^46^ Among the publications from RCTs, 26 investigated G × L,^58^, ^59^, ^60^, ^61^, ^62^, ^63^, ^64^, ^65^, ^66^, ^67^, ^68^, ^69^, ^70^, ^71^, ^72^, ^73^, ^74^, ^75^, ^76^, ^77^, ^78^, ^79^, ^80^, ^81^, ^82^, ^83^ two G × PA,^56^, ^57^ one combined G × PA and G × D,^55^ one combined G × PA and G × L interactions,^54^ and one G × D 53 interactions.

### 3.1 Characteristics of the cohort studies

Eight publications^23^, ^33^, ^34^, ^35^, ^38^, ^39^, ^41^, ^48^ from cohort studies (Table 1) were published from the Nurses' Health Study (NHS) or the Health Professionals Follow‐Up Study (HPFS), six from the Malmö Diet and Cancer study (MDCS),^21^, ^22^, ^26^, ^27^, ^43^, ^45^ five from European Prospective Investigation into Cancer (EPIC)‐InterAct,^14^, ^24^, ^30^, ^40^, ^46^ three from the Shanghai Diabetes GWAS study,^50^, ^51^, ^52^ two each from EPIC‐Potsdam,^19^, ^29^ the Epidemiological Study on the Insulin Resistance Syndrome (DESIR)^25^, ^28^ and the Korean Genome and Epidemiology Study (KOGES),^36^, ^42^ and one each from several other cohort studies.^20^, ^31^, ^32^, ^37^, ^44^, ^47^, ^49^ In NHS and HPFS, the study sample was recruited from the health sector staff and included mainly European descendants.^23^, ^33^, ^34^, ^35^, ^38^, ^39^, ^41^, ^48^ In DESIR, volunteers from health facilities were recruited^25^, ^28^ and in all other studies participants from the general population. The sample size of the studies varied between 718^37^ and 99 166,^31^ with a mean age of participants greater than 40 years in most studies.^14^, ^19^, ^20^, ^21^, ^22^, ^23^, ^24^, ^25^, ^26^, ^27^, ^28^, ^29^, ^30^, ^31^, ^32^, ^33^, ^34^, ^35^, ^36^, ^37^, ^38^, ^39^, ^40^, ^41^, ^42^, ^43^, ^44^, ^45^, ^46^, ^47^, ^48^, ^49^, ^50^, ^51^, ^52^ The follow‐up time ranged from four^32^ to 26 years,^41^ with more than 8 years in most studies.^14^, ^19^, ^20^, ^21^, ^22^, ^23^, ^24^, ^25^, ^26^, ^27^, ^28^, ^29^, ^30^, ^31^, ^32^, ^33^, ^34^, ^35^, ^36^, ^37^, ^38^, ^39^, ^40^, ^41^, ^42^, ^43^, ^44^, ^45^, ^46^, ^47^, ^48^, ^49^, ^50^, ^51^, ^52^ Many studies were conducted in populations of only or mainly European ethnicity.^14^, ^19^, ^20^, ^21^, ^22^, ^23^, ^24^, ^25^, ^26^, ^27^, ^28^, ^29^, ^30^, ^31^, ^33^, ^34^, ^35^, ^37^, ^38^, ^39^, ^40^, ^41^, ^43^, ^44^, ^45^, ^46^, ^47^, ^48^, ^49^ Asians were included in the Shanghai Diabetes study,^50^, ^51^, ^52^ KOGES, and the Korea Association Resource (KARE).^32^, ^36^, ^42^

**Table 1:** Study characteristics of cohort studies

Reference	Study Name	Country (Ethnicity)	Study Type (Samples Recruited from)	No. of Participants (Cases/Total)	Sex	Age (Years)	FU (Years)
Fisher, 2009 19	EPIC‐Potsdam	Germany (European)	Case‐cohort (general population)	724/3042	M, F	35‐65	7.1
Wirström, 2013 20	SDPP	Sweden (European)	Cohort (general population)	178/683	M	∼47, 35‐56	8‐10
Hindy, 2012 21	MDCS	Sweden (European)	Cohort (general population)	1649/24799	M, F	58 ± 7	12
Hindy, 2016 22	MDCS	Sweden (European)	Cohort (general population)	3132/26905	M, F	58 ± 7	14.7
Cornelis, 2009 23	NHS	United States (European desc.)	Case‐control (health professionals)	1140/3055	F	48 ± 7, 30‐55	NR
InterAct Consortium, 2016 24	EPIC‐InterAct	Europe (European)	Case‐cohort (general population)	8086/19121	M, F	51.5	12.5
Li, 2017 14	EPIC‐InterAct	Europe (European)	Case‐cohort (general population)	9937/22273	M, F	ca 55.7, co 52.3	12.2
Lamri, 2012 25	D.E.S.I.R	France (European)	Cohort (volunteers)	191/3646	M, F	47 ± 10, 30‐65	9
Ericson, 2013 26	MDCS	Sweden (European)	Cohort (general population)	1567/24841	M, F	58 ± 7	12
Sonestedt, 2012 27	MDCS	Sweden (European)	Cohort (general population)	1541/24840	M, F	58 ± 8	12
Lamri, 2016 28	D.E.S.I.R	France (European)	Cohort (volunteers)	196/3028	M, F	30‐65	9
Fisher, 2011 29	EPIC‐Potsdam	Germany (European)	Nested case‐control and case‐cohort (general population)	192/576, 614/2862	M, F	35‐65	NR
Li, 2018 30	EPIC‐InterAct	Europe (European)	Case‐cohort (general population)	9742/21900	M, F	ca 55.7, co 52.3	ca 6.8, co 12.3
Bergholdt, 2015 31	CCHS, CGPS, GESUS	Denmark (European)	Cohort (general population)	1355/97811	M, F	20‐100	5.5, (3.7‐7.3)
Lee, 2015 32	KARE	South Korea (Korean)	Cohort (general population)	120/4077 (1128 with prediabetes)	M, F	40‐69	4
Qi, 2009 33	HPFS	United States (European desc.)	Nested case‐control (health professionals)	1196/2533	M	56 ± 8, 40‐75	~14
Beulens, 2007 34	NHS HPFS	United States (European desc.)	Nested case‐control (health professionals)	NHS: 640/1640, HPFS: 383/765	M, F	NHS: 30‐55, HPFS: 40‐75	NR
Cornelis, 2009 35	NHS HPFS	United States (European desc.)	Nested case‐control (health professionals)	NHS: 1612/3775, HPFS: 1297/2909	M, F	NHS: 44 ± 7, HPFS: 55 ± 9	NR
Kim, 2016 36	KoGES	South Korea (Korean)	Cohort (general population)	967/6873	M, F	50.8 ± 8.5, 51.8 ± 8.9	10.0
Song, 2009 37	WHS	United States (mainly European desc.)	Nested case‐control (general population)	359/718	F	≥40	10
Qi, 2005 38	NHS	United States (mainly European desc.)	Nested case‐control (health professionals)	714/1834	F	30‐55	~10
He, 2012 39	NHS HPFS	United States (not described)	Nested case‐control (health professionals)	NHS: 1467/3221, HPFS: 1124/2422	M, F	NHS: 44 ± 7, HPFS: 55 ± 9	NR
Meidtner, 2018 40	EPIC‐InterAct	Europe (European)	Case‐cohort (general population)	9347/21071	M, F	52.7	12.5
Pasquale, 2013 41	NHS HPFS	United States (European desc.)	Nested case‐control (health professionals)	NHS: 1081/2773, HPFS: 725/1998	M, F	NHS: 47.5, HPFS: 54.2	NHS: ~26, HPFS: ~20
Kim, 2017 42	KoGES (Ansan&Ansung)	South Korea (Korean)	Cohort (general population)	984/7024	M, F	40‐69	10
Drake, 2017 43	MDCS	Sweden (European)	Cohort (general population)	2915/20929	M, F	~58	19
Van Hoeck, 2009 44	Rotterdam Study	Netherland (European)	Cohort (general population)	582/6320	M, F	≥55	max 14
Langenberg, 2014 46	EPIC‐InterAct	Europe (European)	Case‐cohort (general population)	12403/28557	M, F	52 ± 9	11.7
Ericson, 2018 45	MDCS	Sweden (European)	Cohort (general population)	3588/25069	M, F	45‐74	17
Brito, 2009 47	Malmö Preventive Project	Sweden (European)	Cohort study (general population)	2063/16003	M, F	~45.5	24.5
He, 2011 48	NHS HPFS	United States (not described)	Nested case‐control (health professionals)	NHS: 1467/3221, HPFS: 1124/2422	M, F	NHS: ~43.5 ± 6.7, HPFS: ~55 ± 8.6	NR
Klimentidis, 2014 49	ARIC	United States (European desc.)	Cohort (general population)	821/8101	M, F	45‐64	7.8
Villegas, 2011 50	SDGS	China (Han Chinese)	Case‐control (general population)	886/2595	F	≤65	NR
Villegas, 2012 51	SDGS, SWHS SMHS	China (Han Chinese)	Case‐control (general population)	2546/5868	M, F	ca: 58.57, co: 53.16	NR
Villegas, 2014 52	SDGS, AGEN‐T2D, SWHS, SMHS	China (Han Chinese)	Case‐control (general population)	Stage I: 886/2595, Stage II: 1647/3347	Stage I: F, Stage II: M,F	Stage I: 50.7, Stage II: 61.66	NR

Abbreviations: AGEN‐T2D, Asian Genetic Epidemiology Network for T2D; ARIC, Atherosclerosis Risk in Communities; CCHS, Copenhagen City Heart Study; CGPS, Copenhagen General Population Study; EPIC, European Prospective Investigation into Cancer and Nutrition; F, female; FU, follow‐up time; GESUS, Danish General Suburban Population Study; HPFS, Health Professionals Follow‐Up Study; IGT; impaired glucose tolerance; KARE, Korean Association Resource; KoGES, Korean Genome and Epidemiology; M, male; MDCS, Malmö Diet and Cancer Study; NHS, Nurses' Health Study; NR, not reported; SDGS, Study Shanghai Diabetes GWAS Study; SDPP, Stockholm Diabetes Prevention Program; SMHS, Shanghai Men's Health Study; SWHS, Shanghai Women's Health Study; WHI‐SHARe, Women's Health Initiative‐SNP Health Association Resource; WHS, Women's Health Study.

### 3.2 Characteristics of the RCTs

Fifteen publications of RCTs were published from the Finish Diabetes Prevention Study (DPS), 14 from the American Diabetes Prevention Project (DPP), one from Prevención con Dieta Mediterránea (PREDIMED),^53^ and one from a study in Italians from Asti.^58^

In DPP (Table 2), 3234 participants with IGT and elevated fasting glucose were randomized into an intensive lifestyle‐intervention group and two standard lifestyle groups with administer of metformin or placebo.^84^ The lifestyle intervention included individual advices and behavior modification to reduce weight by lower fat and calorie intake and higher PA.^84^ In DPS, 522 participants with a high risk for T2D were randomized into a lifestyle‐intervention group or a usual care control group.^85^ The lifestyle‐intervention group was individually guided to reduce weight by increasing PA and following a recommended diet.^85^ In DPP and DPS, the lifestyle intervention resulted in weight reduction and lower T2D risk.^85^, ^86^

**Table 2:** Study characteristics of RCT

Study Name	Country and Ethnicity	Study Population	Treatment Groups	
Finish Diabetes Prevention Study (DPS)	Finland, Europeans	522 healthy, overweight participants (men and women) with IGT, BMI > 25 kg/m^2^, aged 40‐64 years, 66% women, follow‐up ~ 3.2 years	Lifestyle:	weight reduction ≥5%, moderate‐intensity physical activity ≥30 min/day, dietary fat˂30 proportion of total energy (E%), saturated fat ˂ 10 E% or total fat not exceeding 35 E%, and fiber ≥15 g/1000 kcal.
			Controls:	general information about lifestyle and diabetes risk was given individually or in one group session (30 min to 1 h), printed material, no individualized counseling.
American Diabetes Prevention Project (DPP)	USA, European descendants, African‐American, Hispanic, Asian‐American, Indian‐American	3819 healthy participants (men and women) at high risk of developing type 2 diabetes (overweight with elevated fasting glucose, IGT, 68% women age ≥ 25 years, BMI ≥24 kg/m^2^; BMI ≥ 22 kg/m^2^ for Asian‐Americans); 55% were European Ancestry, and 45% were from minority groups; follow‐up 2.8 years	Lifestyle:	moderate‐intensity exercise to achieve and sustain at least 150 min per week of exercise together with a healthy diet to achieve and maintain at least a 7% loss of body weight
			Metformin:	850 mg 2× per day
			Placebo:	standard lifestyle recommendations plus twice‐daily placebo tablets
Asti intervention study	Italy, Europeans	335 participants (mean age 55 years, follow‐up ~4 years, men and women) of a representative cohort of adults from Asti (northwestern Italy) with either the metabolic syndrome or two components of the syndrome and high‐sensitivity C‐reactive protein serum values ≥3 mg/L	Lifestyle: family physician advice and detailed verbal and written individualized recommendations from trained professionals Placebo: standard counseling	
Prevención con Dieta Mediterránea (PREDIMED)	Spain, Europeans	7447 participants (men and women) with either T2D or ≥3 cardiovascular risk factors. Of them, 3671 were nondiabetic participants (mean age 66.6 years, follow‐up time 4.8 years); of the 3671 participants, 286 developed T2D during follow‐up	1) Mediterranean diet supplemented with extra‐virgin olive oil (1 L/week) 2) Mediterranean diet supplemented with mixed nuts (30 g/day) 3) Advice on a low‐fat diet (control diet). Dietary intake was assessed with a validated semiquantitative FFQ and validated 14‐item questionnaire	

In PREDIMED, participants with high cardiovascular risk were randomized into three groups: two Mediterranean‐diet groups with extra‐virgin olive oil (1 L/week) or mixed nuts (30 g/day), and a control group with advice on a low‐fat diet.^53^ In the Italian study,^58^ 335 participants who were nondiabetic and dysmetabolic were randomized into a lifestyle group which received individualized recommendations by trained professionals to reduce metabolic abnormalities and a control group which received standard, unstructured information.

### 3.3 Dietary and PA exposure assessments

**Figure 2:**
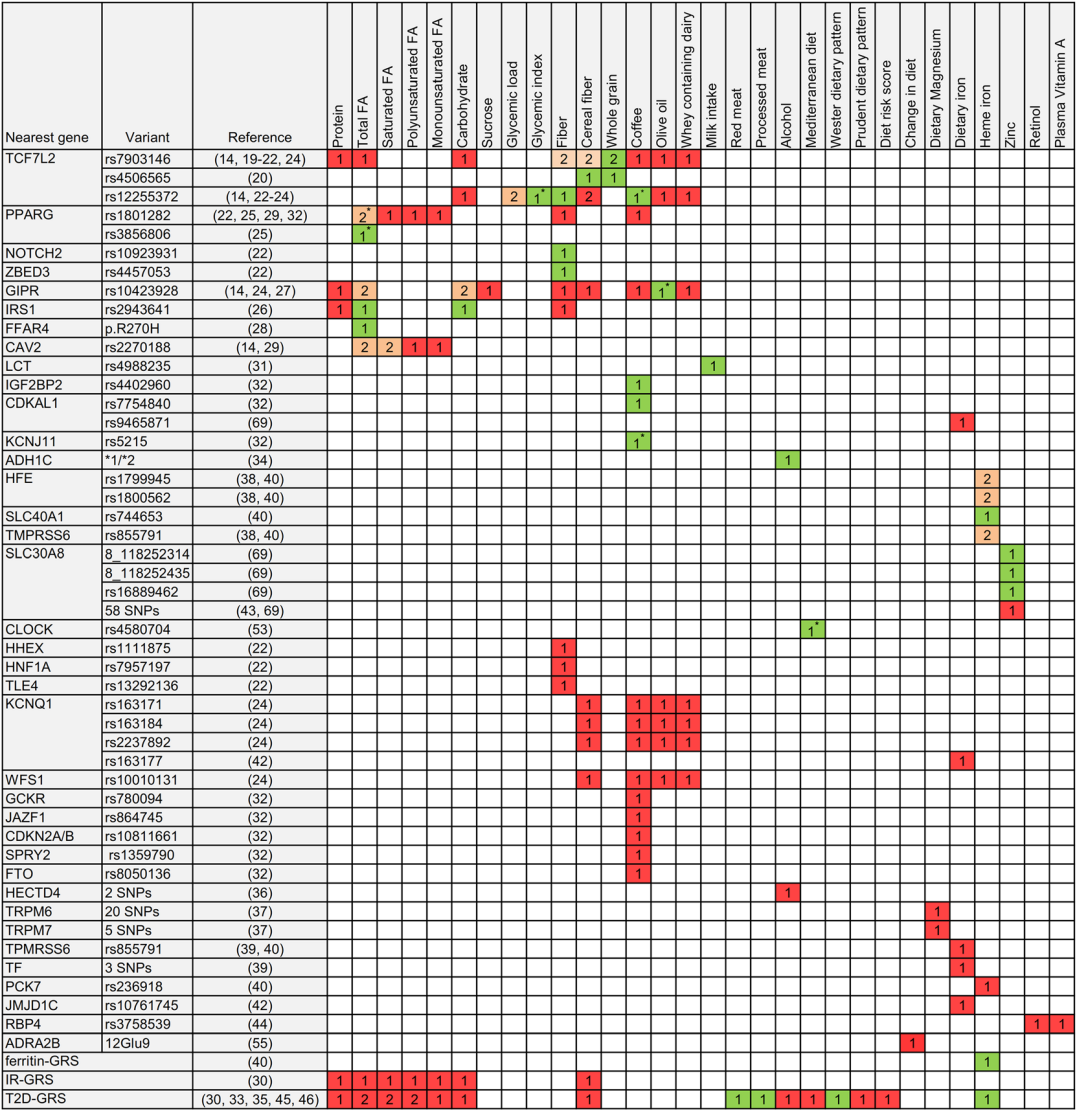
Findings for interaction between genetic variants and diet in relation to T2D incidence. Numbers indicate how many studies investigated the respective gene‐diet interaction, green: interaction was found; red: no interaction was found; orange: contradictory interaction findings. The star sign next to the number indicates that there was only a trend for interaction. Abbreviations: FA, fatty acids; GRS, gene risk score; IR, insulin resistance; T2D, type 2 diabetes mellitus [Colour figure can be viewed at wileyonlinelibrary.com]

The investigated dietary exposure varied considerably across the publications (Figure 2, Table S6) and included individual food groups (whole grain, red meat, olive oil, dairy, and coffee),^19^, ^20^, ^24^, ^31^, ^32^, ^33^ macronutrients (fiber, carbohydrate, fat, and protein),^14^, ^20^, ^21^, ^22^, ^23^, ^24^, ^25^, ^26^, ^27^, ^28^, ^29^, ^35^ micronutrients (magnesium, iron, zinc, and vitamin A),^33^, ^37^, ^38^, ^39^, ^40^, ^41^, ^42^, ^43^, ^44^ alcohol,^34^, ^35^, ^36^ glycemic index and glycemic load,^23^ and dietary patterns.^33^, ^46^, ^53^ Dietary factors were assessed in most studies by validated food frequency questionnaire^14^, ^19^, ^20^, ^21^, ^22^, ^23^, ^24^, ^25^, ^26^, ^27^, ^28^, ^29^, ^31^, ^32^, ^33^, ^34^, ^35^, ^36^, ^37^, ^38^, ^39^, ^40^, ^41^, ^42^, ^43^, ^44^, ^46^, ^53^, ^69^ and in some by dietary history records^14^, ^21^, ^22^, ^24^, ^26^, ^27^, ^40^, ^43^, ^46^ or biomarker measurements.^40^, ^44^ PA (Table S7) was assessed either by in‐person interviews or questionnaires.^46^, ^47^, ^48^, ^49^, ^50^, ^51^, ^52^

### 3.4 Study quality and risk of bias

The NOS quality assessment resulted in high and average quality for 30 and five publications of cohort studies (Table S2), respectively. Reasons for point's deduction were mostly inclusion of selective group of volunteers and health stuffs and no statement for completeness of follow‐up.

The Cochrane risk of bias assessment resulted in low risk of bias for all RCT (Figures S1 and S2). With exception of the performance bias, all judged categories showed low risk of bias. Risk of performance bias was high in all RCT due to the nature of the lifestyle intervention complicating blinding of patient and personal.

The evaluation of methodological quality (Table S3) resulted in high quality for 26 and medium quality for nine cohort study publications. For the RCTs, 13, 17, and one publications were scored as high, medium, and low quality, respectively. Small sample sizes and missing information about Hardy‐Weinberg equilibrium often reduced methodological quality.

### 3.5 Main findings

Of the 66 eligible publications, 19 cohort studies^19^, ^20^, ^21^, ^22^, ^23^, ^24^, ^26^, ^27^, ^28^, ^29^, ^31^, ^32^, ^33^, ^34^, ^38^, ^40^, ^47^, ^48^, ^49^ and nine RCT publications^55^, ^56^, ^57^, ^63^, ^66^, ^69^, ^71^, ^72^, ^74^ reported statistically significant G × E interaction (Figure 2, Tables 3, 4). In addition, some other publications observed significant T2D risk associations in some exposure strata but not in the respective other, although no statistically significant interactions were reported.^25^, ^52^, ^53^, ^59^, ^60^, ^64^, ^68^, ^75^, ^76^ Potential G × D interaction for genetic variants in the *TCF7L2* gene were most frequently investigated, while findings of other genetic variants were often limited to one publication only. Furthermore, publications showed a considerable heterogeneity in investigated dietary factors.

### 3.6 Findings for G × D interaction on incident T2D

#### 3.6.1 Interaction of fiber and whole grain with genetic variants

G × D interactions (Figure 2, Table S6) of *TCF7L2* variants have been the most widely studied so far.^14^, ^19^, ^20^, ^21^, ^22^, ^23^, ^24^ In particular, the *TCF7L2* variant rs7903146 is of interest as it is considered to be strongly associated with T2D risk and known to modify the effect of incretins on insulin secretion. Some publications^19^, ^20^, ^21^, ^22^ reported interaction of *TCF7L2* variants with fiber and whole‐grain intake on T2D incidence (Figure 2, Table S6). Increased cereal fiber intake in the Stockholm Diabetes Prevention Program (SDPP) 20 and fiber intake in MDCS 21 were associated with lower T2D incidence among persons with the rs7903146 CC‐genotype. Contrary, persons with the rs7903146 risk T‐allele showed a slight trend for increased T2D risk with increasing cereal fiber intake in SDPP 20 and with increasing fiber intake in MDCS.^21^, ^22^ An interaction of whole grain with *TCF7L2* rs7903146 was also reported by SDPP^20^ and EPIC‐Potsdam.^19^ In line with the fiber findings, higher whole‐grain intake was in both studies^19^, ^20^ associated with lower T2D incidence among persons with the rs7903146 CC‐genotype, but not among persons with the risk T‐allele. The *TCF7L2* variant rs4506565 also showed interaction with cereal fiber and whole‐grain intake in SDPP^20^ and the *TCF7L2* variant rs12255372 with fiber intake in MDCS.^21^ Contrary, in EPIC‐InterAct and NHS, no interactions of fiber intake with rs7903146 or rs12255372 were observed.^14^, ^23^, ^24^

In MDCS,^22^ fiber intake also showed interactions with the variant rs10923931 in the *NOTCH2* (involved in WNT activity) and rs4457053 in the *ZBED3* (involved in WNT signaling pathway) gene but without replication in another study. Several further variants were investigated in MDCS^22^, ^27^ and in EPIC‐InterAct^24^ but none showed an interaction with fiber (Figure 2).

#### 3.6.2 Interaction of carbohydrates and fat with genetic variants

Interaction of carbohydrates and total fat was reported (Figure 2, Table S6) by MDCS with *IRS1* rs2943641 (known to increase insulin sensitivity) 26 and with G*IPR* rs10423928 (known to decrease insulin secretion).^27^ However, the *IRS1* rs2943641 findings^26^ have not been validated in any other study so far, and the *GIPR* rs10423928 interaction findings could not be replicated in EPIC‐InterAct (12). In NHS,^23^ *TCF7L2* rs12255372 showed no interaction with carbohydrates, but with glycemic load. A replication of this finding in EPIC‐InterAct failed as well.^14^

Potential interaction of fat with genetic variants was also reported by DESIR^25^, ^28^ but without external replication. In DESIR,^25^ a trend for interaction (*P* = .05) was observed between fat intake and the *PPARγ* (receptor for fatty acid storage) variants rs1801282 and rs3856806. Furthermore, H‐allele carriers of the *FFAR4* variant rs116454156 (involved in Gαq signaling) had a fourfold higher T2D incidence in DESIR 28 than RR‐allele carriers but with low fat intake only. In EPIC‐Potsdam,^29^ the *CAV2* variant rs2270188 showed an interaction with total fat and saturated fatty acids but a replicate of the *CAV2* findings in EPIC‐InterAct failed (12). In addition, no evidence was found in EPIC‐InterAct, NHS, and HPFS for interactions of T2D‐GRSs and IR‐GRS with fat and carbohydrates.

#### 3.6.3 Interaction of alcohol with genetic variants

An interaction of alcohol with the ADH1C *1/*2 variant (Figure 2, Table S6) was reported by NHS.^34^ The ADH1C*2‐allele, which is related to a slower rate of ethanol oxidation, attenuated the lower diabetes risk among alcohol drinking US women.^34^ In contrast, such an interaction was not observed among US men.^34^ In addition, a T2D gene risk score (GRS) in NHS and HPFS^35^ and two *HECTD4* variants in KoGES^36^ showed no interaction with alcohol.

#### 3.6.4 Interactions of micronutrients with genetic variants

Findings (Figure 2, Table S6) indicate that the association of heme iron with T2D incidence is modified by a GRS of nine T2D associated loci^33^ and genetic variants of *HFE* (homeostatic iron regulator),^38^, ^40^ *SLC40A1* (iron‐regulated transporter),^40^ and *TMPRSS6* gene (transmembrane serine proteinase).^40^ In HPFS 33, US men with high heme iron intake and high adherence to a T2D‐GRS had a higher T2D risk compared with other exposure strata. In NHS,^38^ higher heme iron intake was associated with increased T2D risk among women with the haemochromatosis‐associated *HFE* variants rs1799945 (H63D) or rs1800562 (C282Y). A similar trend was also observed in EPIC‐InterAct for women with the *HFE* rs1799945 variant, but the corresponding interaction was not significant.^40^ Contrary, for men in EPIC‐InterAct, this interaction was significant.^40^ In EPIC‐InterAct, heme iron intake showed also interaction with the *SLC40A1* rs744653 variant in men and with borderline significance (*P* = .046) for the *TMPRSS6* variant rs855791 in the total sample.^40^ In NHS and HPFS, rs855791 showed no interaction.^39^

For zinc intake, DPP reported an interaction with the *SLC30A8* (zinc transporter) variants rs16889462, 8_118252314, and 8_118252435, but without a clear trend for an association with T2D incidence in exposure strata.^69^ Investigations of potential interaction of some genetic variants with magnesium intake or vitamin A resulted in nonsignificant findings (Figure 2).

#### 3.6.5 Interactions of individual food with genetic variants

Several individual foods have been investigated for potential G × D interaction including red meat, processed meat, olive oil, dairy, and coffee (Figure 2, Table S6). Interactions of red and processed meat with a T2D‐GRS were observed in HPFS.^33^ High intake of red and processed meat increased the risk to develop T2D among men with a high GRS, but not among men with a low GRS.^33^ Olive oil showed a marginally nonsignificant interaction (*P* = .05) with the *GIPR* variant rs10423928 in EPIC‐InterAct.^24^

The association of dairy products with T2D incidence may be modified by the genetic variant rs4988235 near the lactase persistence *LCT* gene.^31^ In a large Danish study, carriers with the lactase persistence *LCT* rs4988235 T‐allele had higher T2D risk compared with persons with the lactose nonpersistence CC‐genotype when they did not consume milk.^31^ A contrasting reduced risk was observed among milk consumers.^31^ Coffee intake is hypothesized to reduce T2D risk, and findings from EPIC‐InterAct^24^ and KARE^32^ indicate that there exist interactions of coffee with some genetic variants. In EPIC‐InterAct, a stronger T2D risk lowering effect with higher coffee intake has been observed for persons with the risk T‐allele of the *TCF7L2* variant rs12255372.^24^ In KARE,^32^ only coffee consumers with the rs4402960 T‐allele (*IGF2BP2*), rs7754840 G‐allele (*CDKAL1*), and rs5215 CC‐genotype (*KCNJ11*) had a reduced combined prediabetes and T2D risk compared with nonconsumer.^32^ Besides these findings, several other genetic variants showed no interaction with coffee, olive oil, or dairy products (Figure 2).

#### 3.6.6 Interactions of dietary patterns with genetic variants

Evidence suggests that dietary patterns that reflect Western diet and enhance T2D incidence and those that reflect Mediterranean diet lower T2D incidence.^87^ In HPFS (Figure 2, Table S6), a Western dietary pattern was associated to higher T2D risk only among persons with a high T2D‐GRS, but not among persons with a low T2D‐GRS.^33^ In PREDIMED,^53^ a borderline significant interaction (*P* = .052) between the *CLOCK* variant rs4580704 and the Mediterranean diet was observed indicating lower T2D incidence among persons with the rs4580704 G‐allele compared with persons with the CC‐genotype.^53^ In EPIC‐InterAct, a Mediterranean‐diet score showed no interaction with a T2D‐GRS.^46^

#### 3.6.7 Findings for G × PA interactions on T2D incidence

Several findings indicate that the protective effect of PA on T2D risk is modified by genetic variants (Tables 3 and S7). From the Atherosclerosis Risk in Communities study, it was reported that the association of PA with lower T2D incidence was weaker among persons with high adherence to a T2D‐GRS compared with persons with a low adherence to the T2D‐GRS.^49^ In the Swedish Malmö preventive program, the minor *HNF1B* rs4430796 A‐allele weakened and possibly reversed the protective effect of higher PA on T2D incidence which was observed in persons with the GG‐genotype.^47^ In NHS, women with the *IRS1* rs1522813 A‐allele and low levels of PA had a higher risk to develop T2D compared with women with the GG‐genotype, but not if they were physically active.^48^

**Table 3:** Findings for interaction between genetic variants and change in leisure time physical activity or physical activity in relation to T2D incidence

*Genetic variants which showed interaction with LTPA in DPS:* *ABCC8* rs3758947; *ADRA2B* 12Glu9; *PPARG* rs17036314, rs1801282; *SLC2A2* rs5393, rs5394, rs5404; *Loci of genetic variants which showed no interaction with LTPA in DPS:* *ABCC8* (3), *ADRB2* (1), *ADRB3* (1), *GHRL* (6), *IGF1R* (1), *IL6* (1), *KCNJ11* (1), *LEPR* (3), *LIPC* (1), *TNF* (1), *PPARG* (1), *SLC2A2* (1)
*Genetic variants which showed interaction with PA in cohort studies:* *HNF1B* rs4430796^a^, *IRS1* rs1522813^b^, T2D‐GRS (65)^c^, IR‐GRS (SNPs of four genes)^c^, FI‐GRS (SNPs of nine genes)^c^ *Loci of genetic variants which showed no interaction with PA in cohort studies:* *PPARD* (9)^d^, *PPARGC* (9)^d^, T2D‐GRS (14)^d^, T2D‐GRS (36)^d^, T2D‐GRS (SNPs of 49 genes)^e^, FG‐GRS (36)^c^, BC‐GRS (SNPs of nine genes)^c^, 65 T2D SNPs^c^, 16 T2D SNPs^a^

Numbers in brackets indicate the numbers of investigated SNPs.

a Malmö Preventive Project.

b Nurses' Health Study/Health Professionals Follow‐Up Study.

c Atherosclerosis Risk in Communities study.

d Shanghai Diabetes GWAS Study.

e InterAct.

Abbreviations: BC, beta cells; DPS, Diabetes Prevention Study; FG, fasting glucose; FI, fasting insulin; GRS, gene risk score; IR, insulin resistance; LTPA, leisure‐time physical activity; PA, physical activity; SDGS, Shanghai Diabetes GWAS Study; T2D, type 2 diabetes mellitus.

In DPS, reduced leisure‐time physical activity (LTPA) (Table 3, S8), controlled for weight and diet changes, was associated with higher T2D incidence among persons with the *PPARγ* rs1801282 Ala‐ and rs17036314 C‐alleles compared with persons with the ProPro‐ and GG‐genotypes.^54^ Contrary, an increase of LTPA resulted in lower T2D incidence among persons with the *ADRA2B* Glu12‐allele, with the *ABCC8* rs3758947 GG‐genotype and with the nonrisk haplotype of the four *SLC2A2* variants rs5393, rs5394, rs5400, and rs5404, whereas persons with the risk allele seem to be unresponsive to change in LTPA.^55^, ^57^ Several further investigated genetic variants showed no interaction with PA or change in LTPA (Table 3).

#### 3.6.8 Findings for G × L interactions on incident T2D

Findings of DPP and DPS indicate that interaction between weight loss interventions to reduce diabetes risk and genetic variants may modify the risk to develop T2D (Tables 4 and S9). In both studies, participants with the *TCF7L2* rs12255372 risk TT‐genotype showed lower T2D incidence in the intervention group than in the control group.^59^, ^60^ Similar findings were reported for persons with the rs7903146 risk T‐allele by DPP and the Asti study.^58^, ^59^ However, the corresponding interaction tests were not significant.^58^, ^59^, ^60^

**Table 4:** Findings for interaction between genetic variants and lifestyle intervention for weight reduction in relation to T2D incidence

*Genetic variants which showed interaction in DPS:* *ADRA2B* 12Glu9, *LIPC* rs2070895, *PPARG* rs1152003, *TNFα* rs1800629, and with trend *CDKN2A/B* rs10811661 *Loci of genetic variants which showed no interaction in DPS:* *ABCG8* (1), *ADAMTS9* (1), *ADIPOQ* (5), *ADIPOR2* (3), *ADRB3* (1), *CDC123* (1), *CDKN2B* (1), *FTO* (1), *IGF2BP2* (1), *IL‐6* (1), *JAZF1* (1), *KCNQ1* (1), *LEPR* (3), *MTNR1B* (1), *NOTCH2* (1), *PPARG* (1), *THADA* (1), *TSPAN8* (1), GRS based on 19 SNPs of T2D‐associated loci
*Genetic variants which showed interaction in DPP:* *ADIPOR2* rs758027*, ENPP1* rs1044498, *MC4R* rs17066829 and with trend *ADIPOQ* rs17373414 *Loci of genetic variants which showed no interaction in DPP:* *ABCC8* (83), *ACE* (1), *ADIPOQ* (19), *ADIPOR1* (22), *ADIPOR2* (26), *ATM* (1), *CAPN10* (33), *CASQ1* (2), *CDKAL1* (1), *CREB1* (13), *CYP3A4* (15), *EXT2* (3), *FOXO1* (34), *GCG* (13), *GCK* (37), *GCKR* (3), *HNF1A* (20), *HNF1B* (73), *HNF4A* (66), *IRS1* (2), *IGF2BP2* (1); *ITLN1* (11), *ITLN2* (15), *KCNJ11* (9), *KCNQ1* (1), *LIPC* (1), *LOC387761* (1), *MC4R* (21), *MEF2A* (38), *MEF2D* (16), *NEUROD1* (14), *NOS3* (6)*, PCK1 (37), PCK2 (14), PDX1* (13), *PKLR* (10), *PPARA* (63), *PPARG* (59), *PRKAG3* (11), *PPARGC1A* (79), *PPARGC1B* (96), *PRKAA1* (9), *PRKAA2* (18), *PRKAB1* (10), *PRKAB2* (13), *PRKAG1* (7), *PRKAG2* (53), *PTPN1* (27), *SLC22A1* (47), *SLC22A2* (44), *SLC30A8* (61), *SLC47A1* (29); *STK11* (10), *TNF* (1), *WFS1* (2), GRS based on 34 T2D‐associated loci
*Genetic variants which showed no interaction in DPS and DPP:* *ADIPOQ* (5), *ADIPOR2* (5), *CDKAL1* rs7754840, *HHEX* rs1111875, *HNF1B* rs757210, *KCNJ11* rs5219^a^ *; PPARG* rs1801282; *SLC30A8* rs13266634; *TCF7L2* rs7903146^a^, rs12255372^a^, *WFS1* rs10010131

Numbers in brackets indicate the numbers of investigated SNPs.

a Variants which showed no interaction but different associations in lifestyle strata.

Abbreviations: DPP, Diabetes Prevention Program; DPS, Diabetes Prevention Study; GRS, gene risk score; T2D, type 2 diabetes mellitus.

Other genetic variants have been investigated only in one RCT study populations, respectively. In DPP, the weight loss intervention was associated with a reduced T2D incidence among persons with the diabetogenic variant of *ENPP1* rs1044498,^72^ and a trend for interaction was found for *MC4R* rs17066829.^73^ Reported interaction findings from DPS^54^, ^66^, ^71^, ^74^ indicate that the weight loss intervention resulted in a lower T2D incidence among persons with the *PPARγ* rs1152003 CC‐genotype,^54^ the *TNFα* rs1800629 GG‐genotype,^71^ the *ADRA2B* Glu9‐allele,^66^ and the *LIPC* rs2070895 A‐allele^74^ compared with the reference genotypes. Both studies investigated also several further genetic variants, but of them none showed an indication for interaction with weight loss intervention (Table 4).

## 4 DISCUSSION

This is the first systematic review summarizing the comprehensive evidence of G × E interactions regarding T2D incidence. Of the 70 eligible publications, 28 publications reported statistically significant G × E interactions. However, the synthesis of findings is limited by the heterogeneity of the investigated genetic variants and dietary exposures. Variants of the *TCF7L2* gene were most frequently investigated and showed potential interactions with whole‐grain and fiber intake, although not consistent across all studies. Other G × D interactions were reported for, eg, a western dietary pattern with a T2D‐GRS, fat and carbohydrate with *IRS1* rs2943641, and heme iron with variants of *HFE*. G × E Interactions were also reported for PA with *HNF1B*, *IRS1*, *PPARγ*, *ADRA2B*, *SLC2A2*, and *ABCC8* variants and for weight loss interventions with *ENPP1*, *PPARγ*, *ADIPOR2*, *ADRA2B*, *TNFα*, and *LIPC* variants. However, the evidence of the reported interactions is hitherto limited as most findings were observed in only one study, mostly of European ethnicity, without validation and replication across multiple study populations.

Findings from EPIC‐InterAct demonstrated that replication of interaction findings poses a major challenge. Surprisingly, none of the previous interaction findings for variants of *TCF7L2*, *GIPR*, *CAV2*, and *HFE* gene with various dietary factors could be confirmed in EPIC‐InterAct.^14^, ^40^ One possible reason for this discrepancy might be false‐positive findings due to noncorrection for multiple hypothesis testing in initial studies.^10^, ^14^ Indeed, to minimize the false‐positive rate, a correction for multiple testing becomes necessary as soon as testing is applied to more than one interrelated hypothesis in the same study sample.^88^ Interactions loosing significance after such a correction include those of *TCF7L2* variant rs7903146 with fiber intake^22^ and of the *CAV2* rs2270188 variant with fat intake.^29^ However, in this context, it should be noted that the correction of *P* values may also cause true interactions to be obscured, especially if the study population is small, and many exposures are tested hypothesis‐free. With regard to the potential interaction of *TCF7L2* variants with whole‐grain or fiber intake, false‐positive findings are an unlikely explanation for the lack of reproducibility in EPIC‐InterAct given that such interaction has been observed in some populations without a multiple testing problem.^19^, ^20^

Another issue in replication of G × E interaction findings concerns exposure measurement errors and reporting bias due to self‐reports of dietary intake and PA. This may introduce variations of measured exposures across studies, and in consequence interaction findings may be distorted and thus consistent replication of interaction findings is hampered.^89^ With regard to the discrepancy between EPIC‐InterAct and earlier studies on the interaction between whole‐grain or fiber intake and *TCF7L2*, it is noteworthy that measurement error is a potential explanation. That the commonly observed inverse association between cereal fiber and diabetes risk has also not been detected in EPIC‐InterAct 24 may reflect an issue to measure this exposure rather than the absence of a true association. Such a measurement error problem would largely affect also the ability to detect differences according to genetic strata.

An appropriate sample size to avoid statistical underpowered analysis is another important issue in G × E interaction research.^10^, ^89^ Low statistical power reduces the chance of detecting a true interaction and thus may produce false negative findings. Genotyping errors, allele frequency, precision of environmental exposure and outcome measurement, and the strength of associations are relevant to determine an adequate statistical power.^89^ A simulation study with underlying RCT design stated that the sample size to detect interaction between two binary exposures is fourfold that to detect a main effect of the same magnitude.^90^ Indeed, it was estimated that more than 30 000 participants are needed to detect an interaction effect of 1.5 with 95% power at a significance level of 10^−4^.^89^ Half of the reviewed cohort study publications included study populations of smaller size (n < 5000), and none of the RCT involved more than 4000 participants. In particular, the DPS has a small sample size and thus limited statistical power to evaluate interactions. While some statistically significant interactions^54^, ^55^, ^57^, ^66^, ^71^, ^74^ were observed by DPS, numerous nonsignificant findings^56^, ^60^, ^62^, ^64^, ^65^, ^67^, ^75^, ^79^, ^82^ have been published. This problem even further exaggerates given that both DPS and DPP have evaluated a large number of genetic variants for interaction with the lifestyle intervention which would require adjustment for multiple testing. Indeed, if one would adjust *P* values of the few reported significant interactions in DPS 54, 55, 57, 66, 71, 74 for all tested variants, none of the interactions would remain significant. In addition, some genetic variants (eg, rs4988235 [near *LCT*], rs17066829 [*MC4R*]), which display significant interactions with lifestyle, have previously not been identified to be associated with T2D incidence in GWAS studies. This may indicate that the effect on T2D risk emerges only as consequence of the interaction between the respective genetic variant and the respective lifestyle factor. Of note is also that DPP often applied interaction tests for three study groups: weight loss intervention, placebo, and also Metformin which could have masked potential interaction.

Although some findings are subject to statistical uncertainty and others are unconfirmed, several of the findings suggest that interactions between genetic variants and the modifiable lifestyle factors diet, PA, and weight loss may modify the risk to develop T2D. Findings indicate that a Western diet high in meat intake enhances the risk to develop T2D especially for individuals with higher genetically susceptibility for T2D.^33^ Meat intake and its components (eg, iron) alone have been linked to higher T2D risk,^91^, ^92^ and it is feasible that genetic variants associated, eg, with impaired insulin sensitivity strengthen this T2D risk effect. Furthermore, a diet rich in fiber and whole grains seems to be protective regarding T2D incidence especially for people with the nonrisk alleles of *TCF7L2*, while those with risk alleles benefit less.^19^, ^20^, ^22^ Fiber‐rich diet leads to a longer retention time of food and is assumed to reduce intestinal glucose absorption, stimulate gastrointestinal hormone secretion, and modulate inflammatory cytokines.^93^ This beneficial effect may be diminished in carriers of the *TCF7L2* rs7903146 T‐risk allele, which has been linked to ß‐cell dysfunction, attenuated insulin secretion and incretin effects, and enhanced rate of hepatic glucose production.^94^ On the other hand, it seems that individuals with the *TCF7L2* risk alleles can reduce their enhanced T2D risk by weight loss due to a healthier lifestyle.^58^, ^59^, ^60^ Further RCT findings suggest as well that individuals with other T2D risk variants may in particular profit from weight loss compared with individuals, which are less genetically susceptible for T2D.^66^, ^71^, ^72^, ^73^, ^74^ Reduced weight seems to mitigate the negative consequences of some T2D risk variants. G × PA interaction findings indicate that individuals at high genetic risk for T2D may profit less from the protective effects of PA on T2D risk than individuals at low genetic risk for T2D.^49^, ^55^, ^57^ Nevertheless, for some T2D risk variants, it was shown that affected individuals can also lower their enhanced T2D risk if they are more physically active.^48^, ^54^ So far, it remains unclear whether these modifying T2D risk effects are attributable to inferior insulin secretion, insulin sensitivity, and ß‐cell dysfunctions in those individuals. In summary, however, there is still a considerable need for research to validate previous results, but also to explore findings in more detail to better understand their pathophysiological impact on T2D risk.

Of note, a large proportion of current evidence rely on study samples with European ethnicities resulting in an underrepresentation of other ethnicities. This is of importance as genetic variants occur with varying frequency in the genome depending on ethnicity. For example, the *TCF7L2* rs7903146 variant is quite common in European ethnicities but not in Asians.^95^ On the contrary, T2D‐associated variants of *KCNQ1* have been found to be more common in Asians than in Danes.^96^ Accordingly, T2D risk modifying effects of G × E interactions in non‐European ethnicities and whether current findings from studies with European ethnicities are transferable to other ethnicities are of high interest for future research.

### 4.1 Strengths and limitations

A strength of the present review is that a comprehensive overview of G × E interaction research regarding T2D incidence is given by including the most important modifiable T2D risk factors diet, PA, and weight loss. Moreover, the focus on prospective studies strengthens the evidence in terms of temporal sequence between G × E interaction and T2D risk development. Finally, this review assessed risk of bias and specific methodological quality of included studies, which mostly showed intermediate and high quality. However, a tool, which is specifically tailored to G × E interaction studies and evaluates both the risk of bias and methodological quality, is not available, to date. The development of such a specific tool should be prioritized in future. Substantial heterogeneity was found with respect to the reported genetic variants and dietary factors, which inhibited meta‐analysis of findings and an evaluation of a possible publication bias. Although our search strategy identified several publications with significant but also nonsignificant interaction findings, a publication bias due to the preferred reporting of significant findings cannot be excluded. In addition, the generalizability of the current evidence was hampered by mostly single study findings and restriction of weight loss interventions to individuals under high risk. Moreover, our systematic review is restricted to the lifestyle factors diet, PA, and weight loss intervention only. Indeed, interactions with genetic variants have also been reported for other lifestyle factors. However, the three reviewed lifestyle factors are considered as main risk factors for T2D.

## 5 CONCLUSION

Although several studies reported gene‐lifestyle interactions, the strength of evidence for modifying effects of these interactions on T2D incidence is still weak. Most of the interaction findings have not yet been replicated across multiple study populations. So far, only interactions of *TCF7L2* rs7903146 with whole grain have been consistently replicated in more than one independent study. A large number of other potential interactions have to be validated first in order to strengthen their evidence. Other potential interactions may be obscured due to limited statistical power. Further analysis in large‐scale studies and formation of collaborative project will possibly bring clarity. However, gene‐environment meta‐analyses across several collaborating studies may be powerful but are very likely to be confronted with issues of comparability and quality of exposure measures. Hence, a prerequisite for such projects is well‐validated, accurate, and precise measured exposures in collaborative studies. Moreover, usage of repeated exposure measurements would also be desirable to facilitate temporal inference. As T2D is a complex, multicausal disorder, limited heritability may be explained by individual variants. The ensemble of genetic variants to form genetic risk scores may therefore be more appropriate to investigate gene‐lifestyle interactions for T2D incidence and should be used more intensively. Investigation of mediator effects of epigenetic markers, which may influence gene‐lifestyle interactions, may also give new insights in the future.

## CONFLICT OF INTEREST

No conflict of interest was declared.

## FUNDING

This work was funded by the European Union's Seventh Framework Programme for research, technological development, and demonstration under grant agreement no 602068. This work was partly supported by a grant from the German Ministry of Education and Research (BMBF) and the State of Brandenburg (DZD grant 82DZD00302). J. Zheng was funded by the Medical Research Council Epidemiology Unit MC_UU_12015/5 and the European Union's Horizon 2020 research and innovation programme under the Marie Sklodowska‐Curie grant agreement no 701708.

## Supporting information


**Table S1**: Judgment score for methodical quality of studies
**Table S2**: Quality assessment of cohort studies by using the Newcastle‐Ottawa Scale
**Table S3**: Methodical quality assessments of included cohort studies and randomized control trials
**Table S4**: Prospective studies which investigated gene‐lifestyle interaction regarding T2D incidence but did not apply a statistical test for interaction
**Table S5**: Assessment of T2D incidence in cohort studies
**Table S6**: Findings for interaction between genetic variants and diet in relation to T2D incidence
**Table S7**: Findings for interaction between genetic variants and physical activity in relation to T2D incidence
**Table S8**: Findings for interaction between genetic variants and change in leisure‐time physical activity in relation to T2D incidence
**Table S9**: Findings for interaction between genetic variants and lifestyle intervention for weight reduction in relation to T2D incidence
**Figure S1**: Risk of bias summary: review authors' judgements about each risk of bias item for each included intervention study
**Figure S2**: Risk of bias graph: review authors' judgements about each risk of bias item presented as percentages across all included studies
