# Supplementary material for: Gene‐lifestyle interaction on risk of type 2 diabetes: A systematic review
Source: Obes Rev. 2019 Sep 2;20(11):1557–71. doi: 10.1111/obr.12921 (PMC8650574; doi:10.1111/obr.12921)
Supplement: Supplementary file 1 — Table S1: Judgment score for methodical quality of studies Table S2: Quality assessment of cohort studies by using the Newcastle‐Ottawa Scale Table S3: Methodical quality assessments of included cohort studies and randomized control trials Table S4: Prospective studies which investigated gene‐lifestyle interaction regarding T2D incidence but did not apply a statistical test for interaction Table S5: Assessment of T2D incidence in cohort studies Table S6: Findings for interaction between genetic variants and diet in relation to T2D incidence Table S7: Findings for interaction between genetic variants and physical activity in relation to T2D incidence Table S8: Findings for interaction between genetic variants and change in leisure‐time physical activity in relation to T2D incidence Table S9: Findings for interaction between genetic variants and lifestyle intervention for weight reduction in relation to T2D incidence Figure S1: Risk of bias summary: review authors' judgements about each risk of bias item for each included intervention study Figure S2: Risk of bias graph: review authors' judgements about each risk of bias item presented as percentages across all included studies [file OBR-20--s001.docx]

# Supporting Information

**Gene-lifestyle interaction on risk of type 2 diabetes: a systematic review**

Stefan Dietrich^1*^, Simone Jacobs^1*^, Ju-Sheng Zheng^2, 3^, Karina Meidtner^1, 4^, Lukas Schwingshackl^5^, Matthias B Schulze^1, 4, 6^

^1^ Department of Molecular Epidemiology, German Institute of Human Nutrition Potsdam Rehbruecke, Nuthetal, Germany

^2^ MRC Epidemiology Unit, School of Clinical Medicine, University of Cambridge, Cambridge, UK

^3^ School of Life Sciences, Westlake University, Hangzhou, China

^4^ German Center for Diabetes Research (DZD), Germany

^5^ Institute for Evidence in Medicine, Faculty of Medicine and Medical Center - University of Freiburg, Freiburg, Germany.

^6^ University of Potsdam, Institute of Nutritional Sciences, Nuthetal, Germany

* SD and SJ contributed equally to the manuscript

**Address for correspondence**

M. B. Schulze, Department of Molecular Epidemiology, German Institute of Human Nutrition Potsdam-Rehbruecke, Nuthetal, Germany.

E-mail: [mschulze@dife.de](mailto:mschulze@dife.de)

Supplemental Note 1: MESH TERMS

(((((((((macronutrient*) OR (micronutrient*) OR (cook*) OR (meal*) OR (intake) or (consumption) OR (nutrition) OR (nutrient*) OR (diet*) OR (“fat intake”) OR (“fatty acid intake”) OR (“fat quality”) OR (“saturated fat*”) OR (SFA) OR (“trans fat*”) OR (“trans-fat*”) OR (“unsaturated fat*”) OR (“monounsaturated fat*”) OR (MUFA) OR (“polyunsaturated fat*”) OR (PUFA) OR (“animal fat”) OR (“dietary protein”) OR (“plant-based protein”) OR (“animal protein”) OR (carbohydrate*) OR (fibre) OR (fiber) OR (grain*) OR (“glycaemic index”) OR (“glycaemic load”) OR (“glycemic index”) OR (“glycemic load”) OR (meat) OR (nuts) OR (fruit*) OR (vegetable*) OR (alcohol) OR (coffee) OR (“sugar-sweetened beverages”) OR (magnesium) OR (zinc) OR (iron) OR (“vitamin D”) OR (”dietary pattern*”) OR (“dietary habit*”) OR (“dietary change*”) OR (“mediterranean score”) OR (“mediterranean diet”) OR (“traditional pattern”) OR (“conservative pattern”) OR (“prudent pattern”) OR (“western diet”) OR (“western pattern”) OR (“Healthy Eating Index”) OR (“physical activity”) OR (“physically active”) OR (training) OR (fitness) OR (exercise) OR (exercising) OR (“energy expenditure”) OR (sport) OR (sports) OR (walking) OR (hiking) OR (biking) OR (cycling) OR (bicycling) OR (swimming) OR (jogging) OR (“yard work”) OR (gardening) OR (“physical inactivity”) OR (“physically inactive”) OR (sedentary) OR (“TV watching”) OR (VO2max) OR (“aerobic capacity”) OR (“Oxygen Consumption”) OR (“Physical Exertion”) OR (“Endurance training”) OR (“Metabolic Equivalent”) OR (“Life Style”) OR (lifestyle))) OR (("Diet"[Mesh]) OR ("Food Quality"[Mesh]) OR ("Meals"[Mesh]) OR ("Cooking"[Mesh]) OR ("Nutritive Value"[Mesh]) OR ("Dietary Carbohydrates"[Mesh]) OR ("Glycemic Index"[Mesh]) OR ("Dietary Proteins"[Mesh]) OR ("meat"[MeSH]) OR ("Meat Products"[Mesh]) OR ("Dietary Fiber"[Mesh]) OR ("Nuts"[Mesh]) OR ("Fatty Acids"[Mesh]) OR ("Dietary Fats"[Mesh]) OR ("Trans Fatty Acids"[Mesh]) OR ("Dietary Fats, Unsaturated"[Mesh]) OR ("Fatty Acids, Omega-3"[Mesh]) OR ("Fatty Acids, Unsaturated"[Mesh]) OR ("Alcohol Drinking"[Mesh]) OR ("Coffee"[Mesh]) OR ("Magnesium"[Mesh]) OR ("Zinc"[Mesh]) OR ("Vitamin D"[Mesh]) OR ("Diet, Western"[Mesh]) OR ("Diet, Mediterranean"[Mesh]) OR ("Diet, High-Fat"[Mesh]) OR ("Motor Activity"[Mesh]) OR ("Exercise"[Mesh]) OR ("Physical Fitness"[Mesh]) OR ("Sports"[Mesh]) OR ("Oxygen Consumption"[Mesh]) OR ("Physical Exertion"[Mesh]) OR ("Metabolic Equivalent"[Mesh]) OR ("Life Style"[Mesh]) OR (“Food”[Mesh]) OR (“Micronutrients”[Mesh])))) AND ((((((((“gene-environment interaction*”) OR (“gene-nutrient interaction*”) OR (“gene-diet interaction*”) OR (“gene-lifestyle interaction*”) OR (“nutrigenomics”) OR (“nutrigenetics”))) OR ("Gene-Environment Interaction"[Mesh] OR "Nutrigenomics"[Mesh])))) OR (((((((Gene) OR (Genotype) OR (genetic*) OR (SNP) OR (Allele*) OR (polymorphism*) OR (“genetic risk score”) OR (“Genetic Predisposition to Disease*") OR (GWAS) OR (GWA) OR (“genome-wide“)))) OR (("Genes"[Mesh]) OR ("Genotype"[Mesh]) OR ("Genetic Variation"[Mesh]) OR (“Genetic Association Studies"[Mesh]) OR ("Polymorphism, Genetic"[Mesh]) OR ("Polymorphism, Single Nucleotide"[Mesh]) OR ("Gene frequency"[Mesh]) OR ("Alleles"[Mesh]) OR ("Genome-Wide Association Study"[Mesh]) OR ("Genomics"[Mesh]) OR ("Genetic Predisposition to Disease"[Mesh])))) AND ((Interact* OR modif* OR modulat* OR stratif*))))))) AND ((((((("Diabetes Mellitus, Type 2/epidemiology"[Mesh]) OR “Diabetes Mellitus, Type 2/genetics*” [Mesh])))) OR ((„Type 2 diabetes“ OR „diabetes type 2“)))))) AND ((((("Randomized Controlled Trials as Topic"[Mesh]) OR ("Randomized Controlled Trial" [Publication Type]) OR ("Controlled Clinical Trials as Topic"[Mesh]) OR ("Cohort Studies"[Mesh]) OR ("Intervention Studies"[Mesh]) OR ("Case-Control Studies"[Mesh]) OR ("Follow-Up Studies"[Mesh]) OR ("incidence"[MeSH]))) OR (((case-control) OR (case-cohort) OR (Cohort) OR (“Randomized Controlled Trial”) OR (RCT) OR (“clinical trial”) OR (Intervention) OR (longitudinal) OR (Follow-Up) OR (prospective) OR (incidence) OR (incident)))))))

**Supplemental tables**

**Table S1:** Judgment score for methodical quality of studies.

|  | Low quality | Intermediate quality | High quality |
| --- | --- | --- | --- |
| Interaction as primary study goal | No = -1 | Not known = 0 | Yes = 1 |
| Formal test for interaction | No = -1 | Not known or stratified analysies= 0 | Yes = 1 |
| Correction for multiple testing | No = -1 | Not known = 0 | Yes or not necessary = 1 |
| Correction for ethnicity | No = -1 | Not known = 0 | Yes or not applicable=1 |
| Hardy-Weinberg equilibrium | No or not stated = -1 | - | Yes = 1 |
| Group similarity at baseline tested | No = -1 | Not known = 0 | Yes = 1 |
| Sample size | <1000 = -1 | 1000-5000 = 0 | >5000 = 1 |
| Sufficient details of study procedure stated | No = -1 | - | Yes = 1 |

Table S2: Quality assessment of cohort studies by using the Newcastle Ottawa Scale

| Author, year (Reference) | Selection | | | | Comparability | | Outcome | | |  |
| --- | --- | --- | --- | --- | --- | --- | --- | --- | --- | --- |
|  | Representative of exposed cohort | Selection of non-exposed cohort | Valid ascertainment of exposure | Outcome of interest not present at start of study | Control for important factors (age, sex, BMI) | Control for additional factors (smoking, energy/diet) | Assessment of outcome | Adequate duration of follow-up (≥5 years) | Adequacy of follow-up | Score |
| Bergholdt, 2015 [^1^](#_ENREF_1) | yes | yes | no | yes | yes | yes | yes | yes | no | 7/9 |
| Beulens, 2007 [^2^](#_ENREF_2) | no | yes | yes | yes | yes | yes | yes | yes | yes | 8/9 |
| Brito, 2009 [^3^](#_ENREF_3) | yes | yes | yes | yes | yes | no | yes | yes | yes | 8/9 |
| Cornelis, 2009 [^4^](#_ENREF_4) | no | yes | yes | yes | yes | yes | yes | yes | yes | 8/9 |
| Cornelis, 2009 [^5^](#_ENREF_5) | no | yes | yes | yes | yes | yes | yes | yes | yes | 8/9 |
| Drake, 2017 [^6^](#_ENREF_6) | yes | yes | yes | yes | yes | yes | yes | yes | yes | 9/9 |
| Ericson, 2013 [^7^](#_ENREF_7) | yes | yes | yes | yes | yes | yes | yes | yes | yes | 9/9 |
| Ericson, 2018 [^8^](#_ENREF_8) | yes | yes | yes | yes | yes | yes | yes | yes | yes | 9/9 |
| Fisher, 2009 [^9^](#_ENREF_9) | yes | yes | yes | yes | yes | yes | yes | yes | yes | 9/9 |
| Fisher, 2011 [^10^](#_ENREF_10) | yes | yes | yes | yes | yes | no | yes | yes | yes | 8/9 |
| He, 2011 [^11^](#_ENREF_11) | no | yes | no | yes | yes | yes | yes | yes | yes | 7/9 |
| He, 2012 [^12^](#_ENREF_12) | no | yes | yes | yes | yes | yes | yes | yes | yes | 8/9 |
| Hindy, 2012 [^13^](#_ENREF_13) | yes | yes | yes | yes | yes | yes | yes | yes | no | 8/9 |
| Hindy, 2016 [^14^](#_ENREF_14) | yes | yes | yes | yes | yes | yes | yes | yes | no | 8/9 |
| InterAct, 2016 [^15^](#_ENREF_15) | yes | yes | yes | yes | yes | yes | yes | yes | yes | 9/9 |
| Kim, 2016 [^16^](#_ENREF_16) | yes | yes | yes | yes | yes | yes | yes | yes | no | 8/9 |
| Kim, 2017 [^17^](#_ENREF_17) | yes | yes | yes | yes | yes | yes | yes | yes | no | 8/9 |
| Klimentidis, 2014 [^18^](#_ENREF_18) | yes | yes | yes | yes | yes | yes | yes | yes | no | 8/9 |
| Lamri, 2012 [^19^](#_ENREF_19) | no | yes | yes | yes | yes | no | yes | yes | no | 6/9 |
| Lamri, 2016 [^20^](#_ENREF_20) | no | yes | yes | yes | yes | no | yes | yes | no | 6/9 |
| Langenberg, 2014 [^21^](#_ENREF_21) | yes | yes | yes | yes | yes | no | yes | yes | yes | 8/9 |
| Lee, 2015 [^22^](#_ENREF_22) | yes | yes | yes | yes | yes | yes | yes | no | yes | 8/9 |
| Li, 2017 [^23^](#_ENREF_23) | yes | yes | yes | yes | yes | yes | yes | yes | yes | 9/9 |
| Li, 2018 [^24^](#_ENREF_24) | yes | yes | yes | yes | yes | yes | yes | yes | yes | 9/9 |
| Meidtner, 2018 [^25^](#_ENREF_25) | yes | yes | yes | yes | yes | yes | yes | yes | yes | 9/9 |
| Pasquale, 2013 [^26^](#_ENREF_26) | no | yes | yes | yes | yes | no | yes | yes | yes | 7/9 |
| Qi, 2009 [^27^](#_ENREF_27) | no | yes | yes | yes | yes | yes | yes | yes | yes | 8/9 |
| Qi, 2005 [^28^](#_ENREF_28) | no | yes | yes | yes | yes | yes | yes | yes | yes | 8/9 |
| Sonestedt, 2012 [^29^](#_ENREF_29) | yes | yes | yes | yes | yes | yes | yes | yes | yes | 9/9 |
| Song, 2009 [^30^](#_ENREF_30) | yes | yes | yes | yes | yes | yes | yes | yes | yes | 9/9 |
| Van Hoek, 2008 [^31^](#_ENREF_31) | yes | yes | yes | yes | yes | yes | yes | yes | no | 8/9 |
| Villegas, 2012 [^32^](#_ENREF_32) | yes | yes | yes | yes | yes | no | yes | NR | no | 6/9 |
| Villegas, 2011 [^33^](#_ENREF_33) | yes | yes | yes | yes | yes | no | yes | NR | no | 6/9 |
| Villegas, 2014 [^34^](#_ENREF_34) | yes | yes | yes | yes | yes | no | yes | NR | no | 6/9 |
| Wirström, 2013 [^35^](#_ENREF_35) | yes | yes | yes | yes | yes | no | yes | yes | yes | 8/9 |

**Table S3:** Methodical quality assessments of included cohort studies and randomized control trials.

|  | Study | Interaction as primary study goal | Statistical test for interaction | Correction for multiple testing | Correction for ethnicity | Hardy-Weinberg Equilibrium | Test of group similarity at baseline | Sample size | Sufficient study details | Score |
| --- | --- | --- | --- | --- | --- | --- | --- | --- | --- | --- |
| Cohort studies | Bergholdt, 2015 [^1^](#_ENREF_1) | 0 | 1 | 1 | 1 | -1 | 1 | 1 | 1 | 5/8 |
|  | Beulens, 2007 [^2^](#_ENREF_2) | 1 | 1 | 1 | 1 | 1 | 1 | 0 | 1 | 7/8 |
|  | Brito, 2009 [^3^](#_ENREF_3) | 1 | 1 | 1 | 1 | 1 | 1 | 1 | 1 | 8/8 |
|  | Cornelis, 2009 [^4^](#_ENREF_4) | 1 | 1 | -1 | 1 | 1 | 1 | 0 | 1 | 5/8 |
|  | Cornelis, 2009 [^5^](#_ENREF_5) | 1 | 1 | -1 | 1 | 1 | 1 | 1 | 1 | 6/8 |
|  | Drake, 2017 [^6^](#_ENREF_6) | 1 | 1 | 1 | 1 | 1 | 1 | 1 | 1 | 8/8 |
|  | Ericson, 2013 [^7^](#_ENREF_7) | 1 | 1 | -1 | 1 | 1 | 1 | 1 | 1 | 6/8 |
|  | Ericson, 2018 [^8^](#_ENREF_8) | 1 | 1 | 1 | 1 | 1 | 1 | 1 | 1 | 8/8 |
|  | Fisher, 2009 [^9^](#_ENREF_9) | 1 | 1 | 1 | 1 | -1 | 0 | 0 | 1 | 4/8 |
|  | Fisher, 2011 [^10^](#_ENREF_10) | 1 | 1 | -1 | 1 | 1 | 1 | 0 | 1 | 5/8 |
|  | He, 2011 [^11^](#_ENREF_11) | 1 | 1 | -1 | 0 | 1 | 1 | 0 | -1 | 2/8 |
|  | He, 2012 [^12^](#_ENREF_12) | 1 | 1 | 1 | 0 | 1 | 1 | 0 | 1 | 6/8 |
|  | Hindy, 2012 [^13^](#_ENREF_13) | 1 | 1 | -1 | 1 | 1 | 1 | 1 | 1 | 6/8 |
|  | Hindy, 2016 [^14^](#_ENREF_14) | 1 | 1 | 1 | 1 | 1 | 1 | 1 | 1 | 8/8 |
|  | InterAct, 2016 [^15^](#_ENREF_15) | 1 | 1 | 1 | 1 | 1 | 1 | 1 | 1 | 8/8 |
|  | Kim, 2016 [^16^](#_ENREF_16) | 1 | 1 | 1 | 1 | 1 | 1 | 1 | 1 | 8/8 |
|  | Kim, 2017 [^17^](#_ENREF_17) | 1 | 1 | 1 | 1 | 1 | 1 | 1 | 1 | 8/8 |
|  | Klimentidis, 2014 [^18^](#_ENREF_18) | 1 | 1 | 1 | 1 | -1 | 1 | 1 | 1 | 6/8 |
|  | Lamri, 2012 [^19^](#_ENREF_19) | 1 | 1 | 1 | 0 | 1 | 1 | 0 | 1 | 6/8 |
|  | Lamri, 2016 [^20^](#_ENREF_20) | 1 | 1 | 1 | 1 | 1 | 0 | 0 | 1 | 6/8 |
|  | Langenberg, 2014 [^21^](#_ENREF_21) | 1 | 1 | 1 | 1 | 1 | 1 | 1 | 1 | 8/8 |
|  | Lee, 2015 [^22^](#_ENREF_22) | 1 | 1 | -1 | 1 | -1 | 1 | 0 | 1 | 3/8 |
|  | Li, 2017 [^23^](#_ENREF_23) | 1 | 1 | 1 | 1 | 1 | 1 | 1 | 1 | 8/8 |
|  | Li, 2018 [^24^](#_ENREF_24) | 1 | 1 | 1 | 1 | 1 | 1 | 1 | 1 | 8/8 |
|  | Meidtner, 2018 [^25^](#_ENREF_25) | 1 | 1 | 1 | 1 | 1 | 1 | 1 | 1 | 8/8 |
|  | Pasquale, 2013 [^26^](#_ENREF_26) | 1 | 1 | 1 | 1 | -1 | 1 | 0 | 1 | 5/8 |
|  | Qi, 2009 [^27^](#_ENREF_27) | 1 | 1 | 1 | 1 | 1 | 1 | 0 | 1 | 7/8 |
|  | Qi, 2005 [^28^](#_ENREF_28) | 1 | 1 | 1 | 0 | 1 | 1 | 0 | 1 | 6/8 |
|  | Sonestedt, 2012 [^29^](#_ENREF_29) | 1 | 1 | -1 | 1 | 1 | 1 | 1 | 1 | 6/8 |
|  | Song, 2009 [^30^](#_ENREF_30) | 1 | 1 | 1 | 1 | 1 | 1 | -1 | 1 | 6/8 |
|  | Van Hoek, 2008 [^31^](#_ENREF_31) | 1 | 1 | 1 | 0 | 1 | 1 | 1 | 1 | 7/8 |
|  | Villegas, 2012 [^32^](#_ENREF_32) | 1 | 1 | -1 | 1 | 1 | 1 | 1 | 1 | 6/8 |
|  | Villegas, 2011 [^33^](#_ENREF_33) | 1 | 1 | -1 | 1 | 1 | 1 | 0 | 1 | 5/8 |
|  | Villegas, 2014 [^34^](#_ENREF_34) | 1 | 1 | -1 | 1 | 1 | 1 | 1 | 1 | 6/8 |
|  | Wirström, 2013 [^35^](#_ENREF_35) | 1 | 1 | -1 | 1 | -1 | 1 | 1 | 1 | 4/8 |
| Randomized control trials | Billlings, 2014 [^36^](#_ENREF_36) | 1 | 1 | -1 | 1 | 1 | 0 | 0 | 1 | 4/8 |
|  | Bo, 2009 [^37^](#_ENREF_37) | 1 | 1 | 1 | 1 | 1 | 1 | -1 | 1 | 6/8 |
|  | Corella, 2016 [^38^](#_ENREF_38) | 1 | 1 | 1 | 1 | 1 | 1 | 0 | 1 | 7/8 |
|  | De Mello, 2015 [^39^](#_ENREF_39) | 1 | 1 | 1 | 1 | -1 | 1 | -1 | 1 | 4/8 |
|  | Florez, 2006 [^40^](#_ENREF_40) | 1 | 1 | 1 | 1 | 1 | 1 | 0 | 1 | 7/8 |
|  | Florez, 2007 [^41^](#_ENREF_41) | 1 | 1 | 1 | 1 | 1 | 0 | 0 | 1 | 5/8 |
|  | Florez, 2008 [^42^](#_ENREF_42) | 1 | 1 | 1 | 1 | 1 | 0 | 0 | -1 | 4/8 |
|  | Florez, 2012 [^43^](#_ENREF_43) | 1 | 1 | -1 | 1 | -1 | 0 | 0 | 1 | 2/8 |
|  | Florez, 2007 [^44^](#_ENREF_44) | 1 | 1 | 1 | 1 | 1 | 1 | 0 | 1 | 7/8 |
|  | Florez, 2012 [^45^](#_ENREF_45) | 1 | 1 | 1 | 1 | -1 | 0 | 0 | 1 | 4/8 |
|  | Hivert, 2011 [^46^](#_ENREF_46) | 1 | 1 | 1 | 1 | 1 | 0 | 0 | 1 | 6/8 |
|  | Jablonski, 2010 [^47^](#_ENREF_47) | 1 | 1 | 1 | 1 | -1 | 1 | 0 | 1 | 5/8 |
|  | Kilpeläinen, 2007 [^48^](#_ENREF_48) | 1 | 1 | 1 | 1 | 1 | 1 | -1 | 1 | 6/8 |
|  | Kilpeläinen, 2008 [^49^](#_ENREF_49) | 1 | 1 | -1 | 1 | 1 | 1 | -1 | 1 | 4/8 |
|  | Kilpeläinen, 2008 [^50^](#_ENREF_50) | 1 | 1 | 1 | 1 | 1 | 1 | -1 | 1 | 6/8 |
|  | Kubaszek, 2003 [^51^](#_ENREF_51) | 0 | 1 | 1 | 1 | 1 | 0 | -1 | 1 | 4/8 |
|  | Laaksonen, 2007 [^52^](#_ENREF_52) | 1 | 1 | 1 | 1 | 1 | 1 | -1 | 1 | 6/8 |
|  | Lappalainen, 2009 [^53^](#_ENREF_53) | 0 | 1 | 1 | 1 | 1 | 1 | -1 | 1 | 5/8 |
|  | Mather, 2012 [^54^](#_ENREF_54) | 0 | 1 | 1 | 1 | -1 | 1 | 0 | 1 | 4/8 |
|  | Moore, 2009 [^55^](#_ENREF_55) | 1 | 1 | 1 | 1 | 1 | 1 | 0 | 1 | 7/8 |
|  | Moore, 2008 [^56^](#_ENREF_56) | 1 | 1 | 1 | 1 | 1 | 0 | 0 | 1 | 6/8 |
|  | Pan, 2013 [^57^](#_ENREF_57) | 1 | 1 | 1 | 1 | 1 | 0 | 0 | 1 | 6/8 |
|  | Pollin, 2011 [^58^](#_ENREF_58) | 1 | 1 | 1 | 1 | 1 | 0 | 0 | 1 | 6/8 |
|  | Salopuro, 2004 [^59^](#_ENREF_59) | -1 | 0 | 1 | 1 | -1 | 1 | -1 | -1 | -1/8 |
|  | Salopuro, 2005 [^60^](#_ENREF_60) | 0 | 1 | 1 | 1 | 1 | 1 | -1 | 1 | 5/8 |
|  | Siitonen, 2004 [^61^](#_ENREF_61) | -1 | 1 | 1 | 1 | 1 | 1 | -1 | 1 | 4/8 |
|  | Siitonen, 2011 [^62^](#_ENREF_62) | 1 | 1 | 1 | 1 | 1 | 0 | -1 | 1 | 5/8 |
|  | Siitonen, 2011 [^63^](#_ENREF_63) | -1 | 1 | 1 | 1 | 1 | 0 | -1 | 1 | 3/8 |
|  | Todorova, 2004 [^64^](#_ENREF_64) | -1 | 1 | 1 | 1 | 1 | 1 | -1 | 1 | 4/8 |
|  | Uusitupa, 2011 [^65^](#_ENREF_65) | 1 | 1 | 1 | 1 | 1 | 1 | -1 | 1 | 6/8 |
|  | Wang, 2007 [^66^](#_ENREF_66) | -1 | 1 | 1 | 1 | 1 | 1 | -1 | 1 | 4/8 |

**Table S4:** Prospective studies which investigated gene-lifestyle interaction regarding T2D incidence but did not apply a statistical test for interaction

| Author, year (Referenz)  Study name | Lifestyle  exposure | Genetic loci  SNP | Association with incident T2D |
| --- | --- | --- | --- |
| Chan, 2015 [^67^](#_ENREF_67)  WHI-SHARe | magnesium intake (validated FFQ) | 583 SNPs in 17 magnesium-related ion channel genes | OR (95% CI) by magnesium (Mg) strata  African American women  rs6584273 (*CNNM1*): low Mg=0.88 (0.74, 1.06), high Mg=**0.71 (0.59, 0.87)**  Hispanic American women  rs1800467 (*KCNJ11*): low Mg =1.11 (0.66, 1.86), high Mg =**2.50 (1.50, 4.28)**  rs7170784 (*NIPA2*): low Mg =1.07 (0.86, 1.34), high Mg =**0.62 (0.47, 0.80)**  rs8028189 (*NIPA2*): low Mg =1.08 (0.87, 1.35), high Mg =**0.65 (0.50, 0.84)** |
| Lindi, 2002 [^68^](#_ENREF_68)  DPS | Weight loss intervention | *PPARG* rs1801282 | OR (95% CI) for Ala12- vs. Pro12Pro  Controls: **2.36 (1.21, 4.60)**, Intervention: 1.90 (0.70, 5.18) |
| Laukkanen, 2004 [^69^](#_ENREF_69)  DPS | Weight loss intervention | *ABCC8* rs3758947, rs2188966, rs3758953, rs1799859  *KCNJ11* rs5219 | OR (95% CI) for haplotype of *ABCC8* SNPs  Control: GGAA=**2.69 (1.27, 5.73)**, Other=**2.62 (1.28 –5.37)**  Intervention: GGAA=2.05 (0.73, 5.74), Other=1.89 (0.73, 4.93)  No significant association for rs5219 (*KCNJ11*) |
| Laukkanen, 2004 [^70^](#_ENREF_70)  DPS | Weight loss intervention | *Insulin* T-23A,  *IGF-1R* GAG1013GAA,  *PC-1* K121Q,  *IRS1* G972R,  *IRS2* G1057D,  *PI3K* p85α M326I | - |
| Laukkanen, 2005 [^71^](#_ENREF_71)  DPS | Weight loss intervention | *SLC2A2* rs5393, rs5394, rs5400, rs5404;  *GCK* rs1799884;  *TCF1* rs1169288, rs2464196;  *HNF4A* rs1884614, rs2144908, rs1885088;  *GIP* rs2291725;  *GLP-1R* rs6923761, rs1042044 | OR (95%CI) *SLC2A2* rs5393 AA vs. C  Control: **5.56 (1.78, 17.39)**, Intervention: 1.17 (0.35, 3.89)  OR (95%CI) *SLC2A2* rs5394 CC- vs. T  Control: 0.85 (0.25–2.86), Intervention: **4.91 (1.56–15.46)**  OR (95%CI) *SLC2A2* rs5400 CC vs. T  Control: 1.40 (0.42–4.64), Intervention: **3.78 (1.50–9.56)**  OR (95%CI) *SLC2A2* rs5404 GG vs. A  Control: 0.84 (0.25–2.82), Intervention: **5.07 (1.61–15.92)** |
| Mager, 2006 [^72^](#_ENREF_72)  DPS | Weight loss intervention | ghrelin gene rs696217 (Leu72Met) | OR (95% CI) for LeuLeu- vs. Met  Controls: 0.61 (0.28, 1.31), intervention: **0.28 (0.10, 0.79)** |

Table S5: Assessment of T2D incidence in cohort studies.

| **Study Name** | **First author, publication year (Reference)** | **Assessment of incident T2D cases** |
| --- | --- | --- |
| ARIC | Klimentidis, 2014 [^18^](#_ENREF_18) | Cases were identified at follow-up visits if they met any of the following criteria: fasting glucose concentration ≥7.0 mmol/L, non-fasting glucose concentration ≥11.1 mmol/L, reported diabetes medication or diabetes diagnosis. |
| CCHS, CGPS, GESUS | Bergholdt, 2015 [^1^](#_ENREF_1) | Cases were identified by using the national Danish Patient Registry and Danish Causes of Death Registry combined with self-reported information on diabetes, diabetes medication, and measured non-fasting glucose. |
| D.E.S.I.R | Lamri, 2012[^19^](#_ENREF_19), 2016 [^20^](#_ENREF_20) | Cases were identified based on the American Diabetes Association criteria: fasting plasma glucose ≥7.0 mmol/l or treatment for diabetes. |
| EPIC-InterAct | Langenberg, 2014 [^21^](#_ENREF_21) InterAct Consortium, 2016 [^15^](#_ENREF_15)  Li, 2017 [^23^](#_ENREF_23), 2018 [^24^](#_ENREF_24)  Meidtner, 2018 [^25^](#_ENREF_25) | Ascertaining incident cases involved a review of the existing EPIC datasets at each center using multiple sources of evidence including self-report, linkage to disease and drug registers, hospital admissions and mortality data. |
| EPIC-Potsdam | Fisher, 2009 [^9^](#_ENREF_9), 2011 [^10^](#_ENREF_10) | Cases were identified by self-administered questionnaires. All self-reports had been verified by the treating physician. |
| KARE, KOGES | Lee, 2015 [^22^](#_ENREF_22)  Kim, 2016 [^16^](#_ENREF_16), 2017 [^17^](#_ENREF_17) | Cases were identified by OGTT during follow-up sessions according to the American Diabetes Association criteria |
| NHS and HPFS | Qi, 2005 [^28^](#_ENREF_28), 2009 [^27^](#_ENREF_27) Beulens, 2007 [^2^](#_ENREF_2)  Cornelis, 2009 [^4^](#_ENREF_4), 2009 [^5^](#_ENREF_5)  He, 2011 [^11^](#_ENREF_11), 2012 [^12^](#_ENREF_12)  Pasquale, 2013 [^26^](#_ENREF_26) | Cases were defined as self-reported diabetes confirmed by a validated supplementary questionnaire. Before 1998 diagnosis was made on the basis of criteria proposed by the National Diabetes Data Group (NDDG). After 1998 the American Diabetes Association diagnostic criteria were used for diagnosis of diabetes cases. |
| Malmö Preventive Project | Brito, 2009 [^3^](#_ENREF_3) | Cases were ascertained using information collected from hospital records detailing a clinical T2D diagnosis or a fasting plasma glucose value ≥7.0 mmol/l during follow-up sessions. |
| MDCS | Hindy, 2012 [^13^](#_ENREF_13), 2016 [^14^](#_ENREF_14)  Sonestedt, 2012 [^29^](#_ENREF_29)  Ericson, 2013 [^7^](#_ENREF_7), 2018[^8^](#_ENREF_8)  Drake, 2017 [^6^](#_ENREF_6) | Cases were identified by three register: 1) the Diabetes 2000 Registry of Scania 2) the Swedish National Diabetes Registry, and 3) the Malmö HbA1c Registry. National Diabetes Register and the Diabetes 2000 register required a diagnosis by a physician at a hospital according to established diagnosis criteria. For non-hospital diagnosed cases the Malmö HbA1c Registry was used. |
| Rotterdam Study | Van Hoeck, 2009 [^31^](#_ENREF_31) | Cases were identified by OGTT and/or treatment with antidiabetic medication and/or a diagnosis of diabetes as registered by a general practitioner according to the guidelines of the World Health Organization and the American Diabetes Association. |
| SDPP | Wirström, 2013 [^35^](#_ENREF_35) | Cases were identified by OGTT during follow-up sessions. |
| Shanghai Diabetes GWAS (SDGS) | Villegas, 2011 [^33^](#_ENREF_33), 2012 [^32^](#_ENREF_32), 2014 [^34^](#_ENREF_34) | Included were cases with fasting glucose level >125 mg/dL at least twice and diabetes medication. |
| WHS | Song, 2009 [^30^](#_ENREF_30) | Cases were identified by self-reports and subsequently mailed supplementary questionnaires to confirm diabetes symptoms, diagnostic tests, and treatments according to the American Diabetes Association criteria. |

**Table S6:** Findings for Interaction between Genetic Variants and Diet in Relation to T2D Incidence

| Author, year (Referenz)  Study name | Diet  (Assessment method) | Genetic loci  SNP | Interaction  for incident T2D | Association with incident T2D* |
| --- | --- | --- | --- | --- |
| Fisher, 2009 [^9^](#_ENREF_9)  EPIC-Potsdam | Whole grain intake  (validated FFQ) | *TCF7L2*  rs7903146 | rs7903146×whole grain (P=0.016) | ↓ T2D risk among persons with CC-genotype with ↑ whole grain intake,  persons with other alleles were unresponsive |
| Wirström, 2013 [^35^](#_ENREF_35)  SDPP | Whole grains,  cereal fiber  (validated FFQ) | *TCF7L2*  rs7903146  rs4506565 | rs7903146 × whole grain  (p=0.008)  rs4506565 × whole grain  (p=0.008)  rs7903146 × cereal fiber  (p=0.005)  rs4506565 × cereal fiber  (p=0.006) | ↓ T2D risk among persons with rs7903146 CC-genotype or rs4506565 AA with ↑ whole grain or ↑ cereal fiber intake,  persons with other alleles were unresponsive |
| Hindy, 2012 [^13^](#_ENREF_13)  MDCS | Total fat, total CHO, protein, fiber  (dietary histories) | *TCF7L2*  rs7903146 | rs7903146 × fiber  (p=0.049) | ↓ T2D risk among persons with rs7903146 CC-genotype with high fiber intake,  persons with T-allele had overall higher T2D risk which was more pronounced with ↑ fiber intake |
| Hindy, 2016 [^14^](#_ENREF_14)  MDCS | fiber  (dietary histories) | *TCF7L2* rs7903146, rs12255372  *HHEX* rs1111875  *HNF1A* rs7957197  *NOTCH2* rs10923931  *TLE4* rs13292136  *ZBED3* rs4457053  *PPARG* rs1801282, rs13081389 | *TCF7L2* rs7903146 × fiber (p=0.034)  *TCF7L2* rs12255372× fiber (p=0.005)  *NOTCH2* rs10923931× fiber (p= 0.017)  *ZBED3* rs4457053 × fiber (p=0.002) | Trend for ↓ T2D risk among persons with rs7903146 CC-genotype and rs12255372 GG-genotype with ↑ fiber intake  Trend for ↑ T2D risk among persons with rs7903146 and rs12255372 T-risk-allele with ↑ fiber intake  ↓ T2D risk among persons with *NOTCH2* rs10923931 and *ZBED3* rs4457053 risk alleles with ↑ fiber intake |
| Cornelis, 2009 [^4^](#_ENREF_4)  NHS | GI, GL, cereal fiber, %CHO  (validated FFQ) | *TCF7L2*  rs12255372 | G × GL  (p=0.03)  trend for G × GI (p=0.06) | ↑ T2D risk among persons with rs12255372 T-risk allele with ↑ tertiles of GL or GI |
| The InterAct Consortium, 2016 [^15^](#_ENREF_15)  EPIC-InterAct | cereal fiber, whey-containing dairy, coffee and olive oil  (validated FFQ or dietary histories) | *TCF7L2* rs7903146 rs12255372  *GIPR* rs10423928  *KCNQ1* rs163171 rs163184 rs2237892  *WFS1* rs10010131 | *TCF7L2* rs12255372 × coffee  (p=0.048)  *GIPR* rs10423928 × olive oil  (p=0.05) | ↓ T2D risk with ↑ Coffee intake among persons with rs12255372 risk T-allele  ↓ T2D risk among persons with rs10423928 TT-genotype but ↑ T2D risk among persons with AA-genotypes with ↑ olive oil intake |
| Li 2017 [^23^](#_ENREF_23)  EPIC-InterAct | fiber, glycemic load, TFA, SFA, total CHO  (validated dietary questionnaires or records) | *TCF7L2* rs7903146  rs12255372  *GIPR* rs10423928  *CAV2* rs2270188  *PEPD* rs3786897 | No interaction | - |
| Lamri, 2012 [^19^](#_ENREF_19)  D.E.S.I.R | Fat intake  (validated FFQ) | *PPARG*  rs1801282 (Pro12Ala)  rs3856806 (1431C>T) | Trend for  rs1801282 × fat (p=0.05)  rs3856806 × fat (p=0.05) | ↑ T2D risk with ↑ fat intake among persons with rs1801282 ProPro- and rs3856806 CC-genotype |
| Ericson, 2013 [^7^](#_ENREF_7)  MDCS | Total fat, total CHO  protein, fiber  (dietary histories) | *IRS1*  rs2943641 | sex × rs2943641 × CHO (p=0.01)  sex × rs2943641 × fat (p=0.01) | ↓ T2D risk among women with T-allele in the lowest or middle tertile of CHO intake  ↓ T2D risk among women with TT-allele in the highest tertile of fat intake  ↓ T2D risk among men with CT-allele in the lowest tertile of fat intake  Persons with other alleles were unresponsive |
| Sonestedt, 2012 [^29^](#_ENREF_29)  MDCS | Total fat, total CHO, sucrose, fiber, protein  (dietary histories) | *GIPR*  rs10423928 | rs10423928 × CHO (p=0.001)  rs10423928 × fat  (p=0.002) | ↓ T2D risk per each additional A-allele in the lowest tertile of CHO intake  ↓ T2D risk per each additional A-allele in the highest tertile of fat intake |
| Lamri, 2016 [^20^](#_ENREF_20)  D.E.S.I.R | Dietary fat  % of energy derived from fat  (validated FFQ) | *FFAR4* SNP p.R270H | G × dietary fat  (p=0.04) | Persons with H-allele had a higher T2D risk than persons with the RR-genotype in the low fat intake category only |
| Fisher, 2011 [^10^](#_ENREF_10)  EPIC-Potsdam | total fat, SFA  PUFA, MUFA  (validated FFQ) | 63 SNPs in 32 candidate or T2D loci  (e.g. CAV2 rs2270188  PPARG rs1801282) | *CAV2* rs2270188×total fat (p=0.02)  *CAV2* rs2270188×SFA (p=0.02) | ↑ T2D risk with ↑ TFA or ↑ SFA intake in persons with rs2270188 TT-genotype |
| Li, 2018[^24^](#_ENREF_24)  EPIC-InterAct | Total CHO, total FA, SFA, PUFA, MUFA  Protein, animal and plant protein, dietary fibre | T2D-GRS | No interaction | - |
| Bergholdt, 2015 [^1^](#_ENREF_1)  CCHS, CGPS,  GESUS | milk intake  (self-reported questionnaires) | rs4988235 near *LCT* | rs4988235 × milk  (additive p=0.005)  (dominat p=0.002) | ↑ T2D risk among non-milk drinker with T-allele    Trend for ↓ T2D risk among milk drinker with T-allele |
| Lee, 2015 [^22^](#_ENREF_22)  KARE | Coffee intake  (validated FFQ) | *GCKR* rs780094  *PPARG* rs1801282 *IGF2BP2* rs4402960 *CDKAL1* rs7754840 *JAZF1* rs864745 *CDKN2A/B* rs10811661  *KCNJ11* rs5215  *SPRY2* rs1359790  *FTO* rs8050136 | G × coffee for  IGF2BP2 rs4402960 (p=0.02)  CDKAL1 rs7754840 (p=0.01)  KCNJ11 rs5215  (p=0.06) | ↓ T2D risk among coffee drinkers with the rs4402960 T-allele or G-allele compared with non-coffee drinker |
| Qi, 2009 [^27^](#_ENREF_27)  HPFS | Red meat, processed meat, heme iron  Western-dietary pattern  Prudent-dietary pattern  (validated FFQ) | T2D GRS derived from 10 SNPs in  9 T2D loci | GRS × red meat  (p=0.02)  GRS × processed meat  (p=0.029)  GRS × heme iron  (p=0.0004)  GRS × Western diet  (p=0.02) | ↑ T2D risk among person with high GRS and high red meat, processed meat or heme iron intake or strong western dietary pattern |
| Beulens, 2007 [^2^](#_ENREF_2)  NHS, HPFS | alcohol consumption (validated FFQ) | Polymorphism in the alcohol dehydrogenase 1c (*ADH1C*) gene | G × alcohol  (women: p=0.02)  (men: p=0.41) | ↓ T2D risk among women with ADH1C*1/*1 or ADH1C*1/*2-allele and high alcohol consumption (≥5 g/day) |
| Cornelis, 2009 [^5^](#_ENREF_5)  NHS, HPFS | Alcohol, PUFA, SFA, TFA, cereal fiber  (validated FFQ) | T2D-GRS derived from 10 SNPs in 9 T2D loci | No interaction | **-** |
| Kim, 2016 [^16^](#_ENREF_16)  KOGES | alcohol consumption  (validated FFQ) | *HECTD4* rs2074356, rs11066280 | No interaction | **-** |
| Song, 2009 [^30^](#_ENREF_30)  WHS | Dietary Magnesium (validated FFQ) | *TRPM6* 20 SNPs  *TRPM7* 5 SNPs | No interaction | - |
| Qi, 2005 [^28^](#_ENREF_28)  NHS | Heme iron intake  (validated FFQ) | *HFE* rs1799945 (H63D) *HFE* rs1800562 (C282Y) | G × iron  (p=0.029) | ↑ T2D risk only in third heme-iron quartile among persons with either variant rs1799935 or rs1800562 |
| He, 2012 [^12^](#_ENREF_12)  NHS, HPFS | Dietary iron  (validated FFQ) | *TPMRSS6* rs855791  *TF* rs3811647  *TF* rs1799852  *TF* rs2280673 | No interaction | - |
| Meidtner, 2018 [^25^](#_ENREF_25)  EPIC-Interact | heme iron intake (validated dietary questionnaires, diet records and biomarker measurements) | weighted ferritin-related gene score and  *HFE* rs1799945  *HFE* rs1800562  *PCK7* rs236918  *SLC40A1* rs744653  *TMPRSS6* rs855791 | gene score × iron in men  (p=0.03)  rs1799945 × iron in men (p=0.01)  rs744653 × iron in women (p=0.002)  rs855791 × iron in all (p=0.046) | Slightly higher T2D risk with high heme intake among men with a low gene score compared to men with a high gene score  Interaction of rs1799945 and heme intake in men was associated with similar raised T2D risk for all subgroups  Trend for ↑ T2D risk in women with high heme intake carrying rs744653 ferritin-raising alleles but also non-ferritin-raising alleles  ↑ T2D risk in persons with high heme intake and no rs855791 ferritin-raising alleles |
| Pasquale, 2013 [^26^](#_ENREF_26)  NHS, HPFS | heme iron intake  (validated FFQ) | genome-wide SNPs for iron metabolism | No interaction | - |
| Kim, 2017 [^17^](#_ENREF_17)  KOGES | dietary iron intake (validated FFQ) | *CDKAL1* rs9465871, *JMJD1C* rs10761745,  *KCNQ1* rs163177 | No interaction | - |
| Billings, 2014 [^36^](#_ENREF_36)  DPP | Zinc intake  (validated FFQ) | 61 *SLC30A8*  SNPs | 8_118252314 × zinc (p=0.03)  8_118252435 × zinc (p=0.02)  rs16889462 × zinc (p=0.025) | no significant association in genotype strata |
| Drake, 2017 [^6^](#_ENREF_6)  MDCS | Zinc intake  (diet history, FFQ and 7-day food record) | *SLC30A8* rs13266634 | No interaction | - |
| Van Hoeck, 2009 [^31^](#_ENREF_31)  Rotterdam Study | retinol intake  (validated FFQ)  plasma vitamin A  (biomarker measurements) | RBP4  rs3758539 | No interaction | - |
| Langenberg, 2014 [^21^](#_ENREF_21)  EPIC-InterAct | Mediterranean diet score (validated FFQ) | 49 SNPs of 49 T2D loci  T2D GRS | No interaction | - |
| Corella, 2016 [^38^](#_ENREF_38)  PREDIMED | Mediterranean Diet Intervention | *CLOCK* rs4580704 | Trend for G × D  (p=0.052) | ↓ T2D risk among persons with G-allele in MedDiet group compared to controls |
| Ericson, 2018 [^8^](#_ENREF_8)  MDCS | diet risk score based on intakes of processed meat, sugar-  sweetened beverages, whole grain and coffee  (dietary histories) | GRS based on 48 T2D SNPs | No interaction | - |
| Laaksonen, 2007 [^52^](#_ENREF_52)  DPS | Change in diet  Intervention | ADRA2B  12Glu9 | No interaction | favorable dietary changes ↓ T2D risk only in persons with the Glu9/9-genotype |

*Only most important findings are listed. Abbreviations: AGEN-T2D, Asian Genetic Epidemiology Network for T2D; ARIC, Atherosclerosis Risk in Communities; CCHS , Copenhagen City Heart Study; CGPS, Copenhagen General Population Study; CI, confidence interval; CHO, carbohydrate; DPP, US Diabetes Prevention Program; DPS, Finnish Diabetes Prevention Study; EPIC, European Prospective Investigation into Cancer and Nutrition; F, Female; FFQ, food frequency questionnaires; FG, fasting glucose; FI, fasting insulin; FU, Follow-up time; GESUS, Danish General Suburban Population Study; GI, glycemic index; GL, glycemic load; GRS, gene risk score; HPFS, Health Professionals Follow-Up Study; HR, Hazards ratio; IGT, impaired glucose tolerance; IR, insulin resistence; KARE, Korean Association Resource; KoGES, Korean Genome and Epidemiology; M, Male; MDCS, Malmö Diet and Cancer Study; NHS, Nurses’ Health Study; NR, not reported; PA, physical activity; OR, odds ratio; PREDIMED, Prevención con Dieta Mediterránea, RR, relative risk; SDGS, Study Shanghai Diabetes GWAS Study; SDPP, Stockholm Diabetes Prevention Program; SFA, saturated fat; SMHS, Shanghai Men’s Health Study; SWHS, Shanghai Women’s Health Study; T2D, type two diabetes mellitus; TFA, total fat, WHI-SHARe, Women's Health Initiative-SNP Health Association Resource; WHS, Women’s Health Study.

**Table S7:** Findings for Interaction between Genetic Variants and Physical Activity in Relation to T2D Incidence

| Author, year (Referenz)  Study name | Genetic loci | PA  (Assessment method) | Interaction | Association with incident T2D* |
| --- | --- | --- | --- | --- |
| Brito, 2009 [^3^](#_ENREF_3)  MPP | 17 SNPs of T2D genes e.g. *HNF1B* rs4430796  *PPARG* rs1801282  *SLC30A8* rs13266634  *TCF7L2* rs7903146  *KCNJ11* rs5219 | classified as physical active or inactive  (self-administered computer-based questionnaire) | rs4430796 × PA (p=0.0068) | GG-genotypes showed higher cumulative T2D incidence than A-allele carrier when physical inactive. |
| He, 2011 [^11^](#_ENREF_11)  NHS, HPFS | *IRS1* rs1522813  *IRS1* rs2943641 | low vs. high PA  (metabolic equivalent task) | rs1522813 × PA  (women p=0.017)  men: p=0.26) | Women with the A-allele had ↑T2D risk with low PA compared to women with GG-genotype.  No difference in T2D when PA was high. |
| Klimentidis, 2014 [^18^](#_ENREF_18)  ARIC | 65 T2D associated SNPs  T2D-GRS (65 SNPs)  FG-GRS (36 SNPs)  FI-GRS (17 SNPs)  IR-GRS (SNPs of 4 genes)  BC-GRS (SNPs of 9 genes) | PA index  (Baecke PA questionnaire) | T2D-GRS × PA  (overall p=0.016  women p=0.0025  men p=0.46)  IR-GRS × PA (p=0.046)  FI-GRS × PA (p=0.042) | Person with low T2D-GRS showed trend for decreased T2D risk per 1 unit increase in PA compared to person with high T2D-GRS which were unresponsive regarding T2D risk.  A similar trend was observed for the IR-GRS and FI-GRS |
| Langenberg, 2014 [^21^](#_ENREF_21)  EPIC-InterAct | T2D-GRS of 49 T2D associated loci | PA index  (validated brief questionnaire) | No interaction | - |
| Villegas, 2011 [^33^](#_ENREF_33)  SDGS | PPARD  39 SNPs | PA  (in-person interviews) | No interaction | - |
| Villegas, 2012 [^32^](#_ENREF_32)  SDGS | **GRS1**: (36 SNPs)  **GRS2**: (14 T2D-associated SNPs) | PA patterns  (in-person interviews or validated questionnaires) | No interaction | - |
| Villegas, 2014 [^34^](#_ENREF_34)  SDGS  AGEN-T2D | SNPs of PPAR and PGC1 genes | PA patterns  (in-person interviews or validated questionnaires) | No interaction | Rs1549188 (*PPARGC1B*) was associated with higher T2D risk the non-exercise group only.  Rs251464 (*PPARGC1B*) was associated with lower T2D risk in the non-exercise group only |

*Only most important findings are listed. Abbreviations: AGEN-T2D, Asian Genetic Epidemiology Network for T2D; ARIC, Atherosclerosis Risk in Communities; BC, beta cells; CI, confidence interval; FG, fasting glucose; FI, fasting insulin; GRS, gene risk score; HPFS, Health Professionals Follow-Up Study; IR, insulin resistence; MPP, Malmö Preventive Project; NHS, Nurses’ Health Study; PA, physical activity; T2D, type two diabetes mellitus, SDGS, Shanghai Diabetes GWAS Study;

**Table S8:** Findings for Interaction between Genetic Variants and Change in Leisure Time Physical Activity in Relation to T2D Incidence

| Author, year (Referenz) | Genetic loci | Interaction | Association with incident T2D* |
| --- | --- | --- | --- |
| Kilpeläinen, 2008 [^49^](#_ENREF_49)  DPS | *PPARG* rs17036314,  rs1801282, rs1152003 | rs1801282 × LTPA  (p=0.031)  rs17036314 × LTPA  (p=0.002) | A change to higher LTPA removed the higher T2D risk in person with the rs1801282 and rs17036314 risk alleles. |
| Laaksonen, 2007 [^52^](#_ENREF_52)  DPS | *ADRA2B* 12Glu9 | 12Glu9 × LTPA  (p=0.033) | ↓T2D risk among person with the Glu12-allele with ↑ LTPA.  Person with Glu9/9-genotype were unresponsive to change in LTPA |
| Kilpeläinen, 2008 [^50^](#_ENREF_50)  DPS | SNPs in *ADRB2, ADRB3, TNF, IL6, IGF1R, LIPC, LEPR, GHRL* | No interaction | - |
| Kilpeläinen, 2007 [^48^](#_ENREF_48)  DPS | *SLC2A2* rs5393, rs5394, rs5400, rs5404  *ABCC8* rs3758947, rs2188966, rs3758953, rs1799859  *KCNJ11* rs5219 | G × LTPA for  *SLC2A2* rs5393 (p=0.027)  *SLC2A2* rs5394 (p=0.022  *SLC2A2* rs5404 (p=0.022)  *ABCC8* rs3758947 (p=0.007)  *SLC2A2* CTTA haplotype (p=0.021) | Person with the common homozygous genotype of rs5393, rs5394, or rs5404 of SLC2A2 and rs3758947 of ABCC8 who were in the upper third of the change in LTPA had a lower T2D risk compared to the lower and middle third groups.  Person with the rare allele carriers were unresponsive to change in LTPA. |

*Only most important findings are listed. Abbreviations: CI, confidence interval; DPS, diabetes prevention study; LTPA, Leisure Time Physical Activity; RR, relative risk; T2D, type two diabetes mellitus

**Table S9:** Findings for Interaction between Genetic Variants and Lifestyle Intervention for Weight Reduction in Relation to T2D Incidence

| Author, year (Referenz) | Genetic loci | Interaction | Association with incident T2D* |
| --- | --- | --- | --- |
| Bo, 2009 [^37^](#_ENREF_37)  Asti study | *TCF7L2* rs7903146 | No interaction | Trend for ↑ T2D risk in person with T-allele in control group, but not in intervention group |
| Florez, 2006 [^40^](#_ENREF_40)  DPP | *TCF7L2* rs12255372  rs7903146 | No interaction | ↑ T2D risk among rs12255372 or rs7903146 TT-genotypes in the placebo group, but not in the intervention group |
| Wang, 2007 [^66^](#_ENREF_66)  DPS | *TCF7L2* rs12255372  rs7903146 | No interaction | ↑ T2D risk in controls with rs12255372 TT-genotype but not in intervention group  No significant T2D risk association for rs7903146 in study groups |
| Florez, 2007 [^41^](#_ENREF_41)  DPP | *PPARG* rs1801282 | No interaction | - |
| Kilpeläinen, 2008 [^49^](#_ENREF_49)  DPS | *PPARG* rs17036314,  rs1801282, rs1152003 | rs1152003×T | ↑ T2D risk in intervention group among persons with rs1152003 CC-genotype compared to person with G-allele but not in controls |
| Uusitupa, 2011 [^65^](#_ENREF_65)  DPS | 19 SNPs of T2D genes (e.g. *PPARG* rs1801282  *KCNJ11* rs5219  *TCF7L2* rs7903146,  *SLC30A8* rs13266634,  *FTO* rs9939609) | No interaction | - |
| Mather, 2012 [^54^](#_ENREF_54)  DPP | 77 tagging SNPs  -*ADIPOQ* (24)  -*ADIPOR1* (22)  -*ADIPOR2* (31) | *ADIPOR2* rs758027 × T  *ADIPOQ* rs17373414 × T (included Metformin group) | ↓T2D risk in persons with rs758027 C-allele compared to T-allele in placebo group, intervention group was unresponsive  No significant T2D risk association for rs17373414 in study groups |
| Siitonen, 2011 [^62^](#_ENREF_62)  DPS | *ADIPOQ*  rs266729, rs16861205, rs169861210, rs17366568, rs2241766, rs1501299, rs3821799, rs17366743, rs6773957, rs2082940 | No interaction | - |
| Siitonen, 2011 [^63^](#_ENREF_63)  DPS | *ADIPOR2*  rs10848554, rs11061937, rs11061946, rs1058322, rs11061973, rs4766415, rs16928751, rs1044471 | No interaction | - |
| Siitonen, 2004 [^61^](#_ENREF_61)  DPS | *ADRA2B*  12Glu9 | G × T | ↑ T2D risk in control group among persons with Glu9-allele compared  to persons with the Glu12/12 genotype, but not in the intervention group |
| Salopuro, 2004 [^59^](#_ENREF_59)  DPS | *ADRB3*  Trp64Arg | No interaction | - |
| Florez, 2007 [^44^](#_ENREF_44)  DPP | *KCNJ11* rs5219 (E23K)  *ABCC8* rs757110 (A1369S) | No interaction  (included metformin group) | - |
| Billings, 2014 [^36^](#_ENREF_36)  DPP | 61 *SLC30A8* SNPs | No interaction | - |
| Moore, 2008 [^56^](#_ENREF_56)  DPP | *CDKN2A/B* rs10811661  *HHEX* rs1111875, rs7923837  *EXT2* rs11037909, rs3740878, rs1113132  *SLC30A8* rs13266634  LOC387761 rs7480010  *IGF2BP2* rs1470579  *CDKAL1* rs7754840 | rs10811661 × T  (p=0.05)  (included metformin group) | Trend for attenuated T2D risk per rs10811661 T-allele in intervention group compared to placebo group |
| Kubaszek, 2003 [^51^](#_ENREF_51)  DPS | *TNFα* G-308A  *IL-6* C-174G | G-308A × L | ↓T2D risk in persons with the  *TNFα* GG-genotype compared with A-allele in intervention group but not in control group. |
| Moore, 2009 [^55^](#_ENREF_55)  DPP | *ENPP1*  rs1044498 (K121Q) | G × T  (included metformin group) | ↑ T2D risk in persons with Q allele compared with KK genotype in placebo group, Lifestyle intervention eliminated this increased risk. |
| Pan, 2013 [^57^](#_ENREF_57)  DPP | *MC4R* locus variants (20 SNPs) | rs17066829 × T  (included metformin group) | Trend for ↓T2D risk in intervention group per rs17066829 A-allele but not in controls |
| Todorova, 2004 [^64^](#_ENREF_64)  DPS | *LIPC* G-250A | G×L | ↓T2D risk in persons with A-allele in intervention group compared to GG-genotypes and controls |
| Salopuro, 2005 [^60^](#_ENREF_60)  DPS | *LEPR*  rs1137100 (Lys109Arg)  rs1137101 (Gln223Arg)  3'UTR Del/Ins | No interaction | ↑ T2D risk in persons with rs1137100 LysLys genotype compared with Arg-allele in control group but not in intervention group  ↑ T2D risk in persons with rs1137100 GlnGln genotype compared with Arg-allele in control group but not in intervention group |
| Florez, 2008 [^42^](#_ENREF_42)  DPP | *WFS1* rs10010131,  rs752854, rs734312 | No interaction  (included metformin group) | ↓ T2D risk in Europeans descendants with rs752854 GG-genotype compared with A-allele in intervention group but not in placebo group |
| Florez, 2012 [^45^](#_ENREF_45)  DPP | *ATM* rs11212617 | No interaction  (included metformin group) | - |
| Hivert, 2011 [^46^](#_ENREF_46)  DPP | GRS based on 34 T2D-associated loci | No interaction  (included metformin group) | - |
| De Mello, 2015 [^39^](#_ENREF_39)  DPS | *ABCG8* rs4299376 | No interaction | - |
| Florez, 2012 [^43^](#_ENREF_43)  DPP | *G6PC2, MTNR1B, GCK, DGKB, GCKR, ADCY5, MADD, CRY2, ADRA2A, FADS1, PROX1, SLC2A2, GLIS3, C2CD4B, IGF1, IRS1* | No interaction  (included metformin group) | - |
| Jablonski, 2010 [^47^](#_ENREF_47)  DPP | 1590 SNPs in 40 candidate and T2D loci | No interaction | - |
| Lappalainen, 2009 [^53^](#_ENREF_53)  DPS | *FTO* rs9939609 | No interaction | - |
| Pollin, 2011 [^58^](#_ENREF_58)  DPP | *GCKR* rs780094,  rs1260326 | No interaction | - |

*Only most important findings are listed. Abbreviations: CI, confidence interval; DPP, diabetes prevention program; DPS, diabetes prevention study; GRS, gene risk score; HR, Hazards ratio; NR, not reported; OR, odds ratio; T2D, type two diabetes mellitus

**Supplemental Figures**


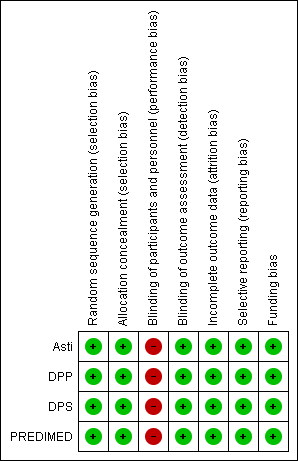


**Figure S1:** Risk of bias summary: review authors' judgements about each risk of bias item for each included intervention study.


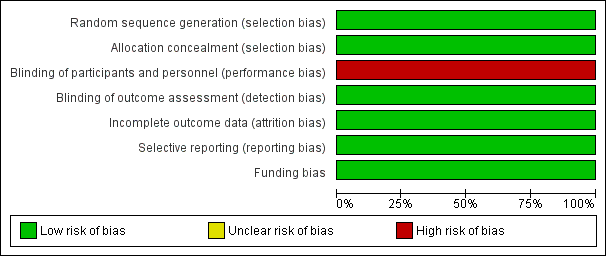
**Figure S2:** Risk of bias graph: review authors' judgements about each risk of bias item presented as percentages across all included studies.

**References**

1 Bergholdt HKM, Nordestgaard BG, Ellervik C. Milk intake is not associated with low risk of diabetes or overweight-obesity: a Mendelian randomization study in 97,811 Danish individuals. *American Journal of Clinical Nutrition*. 2015; 102: 487-96.

2 Beulens JW, Rimm EB, Hendriks HF, Hu FB, Manson JE, Hunter DJ*, et al.* Alcohol consumption and type 2 diabetes: influence of genetic variation in alcohol dehydrogenase. *Diabetes*. 2007; 56: 2388-94.

3 Brito EC, Lyssenko V, Renstrom F, Berglund G, Nilsson PM, Groop L*, et al.* Previously Associated Type 2 Diabetes Variants May Interact With Physical Activity to Modify the Risk of Impaired Glucose Regulation and Type 2 Diabetes A Study of 16,003 Swedish Adults. *Diabetes*. 2009; 58: 1411-18.

4 Cornelis MC, Qi L, Kraft P, Hu FB. TCF7L2, dietary carbohydrate, and risk of type 2 diabetes in US women. *Am J Clin Nutr*. 2009; 89: 1256-62.

5 Cornelis MC, Qi L, Zhang C, Kraft P, Manson J, Cai T*, et al.* Joint effects of common genetic variants on the risk for type 2 diabetes in U.S. men and women of European ancestry. *Ann Intern Med*. 2009; 150: 541-50.

6 Drake I, Hindy G, Ericson U, Orho-Melander M. A prospective study of dietary and supplemental zinc intake and risk of type 2 diabetes depending on genetic variation in SLC30A8. *Genes & nutrition*. 2017; 12: 30.

7 Ericson U, Rukh G, Stojkovic I, Sonestedt E, Gullberg B, Wirfalt E*, et al.* Sex-specific interactions between the IRS1 polymorphism and intakes of carbohydrates and fat on incident type 2 diabetes. *American Journal of Clinical Nutrition*. 2013; 97: 208-16.

8 Ericson U, Hindy G, Drake I, Schulz CA, Brunkwall L, Hellstrand S*, et al.* Dietary and genetic risk scores and incidence of type 2 diabetes. *Genes & nutrition*. 2018; 13: 13.

9 Fisher E, Boeing H, Fritsche A, Doering F, Joost H-G, Schulze MB. Whole-grain consumption and transcription factor-7-like 2 (TCF7L2) rs7903146: gene-diet interaction in modulating type 2 diabetes risk. *British Journal of Nutrition*. 2009; 101: 478-81.

10 Fisher E, Schreiber S, Joost H-G, Boeing H, Doering F. A Two-Step Association Study Identifies CAV2 rs2270188 Single Nucleotide Polymorphism Interaction with Fat Intake in Type 2 Diabetes Risk. *Journal of Nutrition*. 2011; 141: 177-81.

11 He MA, Workalemahu T, Cornelis MC, Hu FB, Qi L. Genetic variants near the IRS1 gene, physical activity and type 2 diabetes in US men and women. *Diabetologia*. 2011; 54: 1579-82.

12 He M, Workalemahu T, Manson JE, Hu FB, Qi L. Genetic Determinants for Body Iron Store and Type 2 Diabetes Risk in US Men and Women. *Plos One*. 2012; 7.

13 Hindy G, Sonestedt E, Ericson U, Jing XJ, Zhou Y, Hansson O*, et al.* Role of TCF7L2 risk variant and dietary fibre intake on incident type 2 diabetes. *Diabetologia*. 2012; 55: 2646-54.

14 Hindy G, Mollet IG, Rukh G, Ericson U, Orho-Melander M. Several type 2 diabetes-associated variants in genes annotated to WNT signaling interact with dietary fiber in relation to incidence of type 2 diabetes. *Genes & nutrition*. 2016; 11: 6.

15 The InterAct Consortium. Investigation of gene-diet interactions in the incretin system and risk of type 2 diabetes: the EPIC-InterAct study. *Diabetologia*. 2016; 59: 2613-21.

16 Kim J, Oh B, Lim JE, Kim MK. No Interaction with Alcohol Consumption, but Independent Effect of C12orf51 (HECTD4) on Type 2 Diabetes Mellitus in Korean Adults Aged 40-69 Years: The KoGES_Ansan and Ansung Study. *PLoS One*. 2016; 11: e0149321.

17 Kim J, Kim MK, Jung S, Lim JE, Shin MH, Kim YJ*, et al.* Interaction of iron status with single nucleotide polymorphisms on incidence of type 2 diabetes. *PLoS One*. 2017; 12: e0175681.

18 Klimentidis YC, Chen Z, Arora A, Hsu C-H. Association of physical activity with lower type 2 diabetes incidence is weaker among individuals at high genetic risk. *Diabetologia*. 2014; 57: 2530-34.

19 Lamri A, Khalil CA, Jaziri R, Velho G, Lantieri O, Vol S*, et al.* Dietary fat intake and polymorphisms at the PPARG locus modulate BMI and type 2 diabetes risk in the D.E.S.I.R. prospective study. *International Journal of Obesity*. 2012; 36: 218-24.

20 Lamri A, Bonnefond A, Meyre D, Balkau B, Roussel R, Marre M*, et al.* Interaction between GPR120 p.R270H loss-of-function variant and dietary fat intake on incident type 2 diabetes risk in the D.E.S.I.R. study. *Nutr Metab Cardiovasc Dis*. 2016; 26: 931-6.

21 Langenberg C, Sharp SJ, Franks PW, Scott RA, Deloukas P, Forouhi NG*, et al.* Gene-Lifestyle Interaction and Type 2 Diabetes: The EPIC InterAct Case-Cohort Study. *Plos Medicine*. 2014; 11.

22 Lee JK, Kim K, Ahn Y, Yang M, Lee JE. Habitual coffee intake, genetic polymorphisms, and type 2 diabetes. *European Journal of Endocrinology*. 2015; 172: 595-601.

23 Li SX, Imamura F, Ye Z, Schulze MB, Zheng J, Ardanaz E*, et al.* Interaction between genes and macronutrient intake on the risk of developing type 2 diabetes: systematic review and findings from European Prospective Investigation into Cancer (EPIC)-InterAct. *Am J Clin Nutr*. 2017; 106: 263-75.

24 Li SX, Imamura F, Schulze MB, Zheng J, Ye Z, Agudo A*, et al.* Interplay between genetic predisposition, macronutrient intake and type 2 diabetes incidence: analysis within EPIC-InterAct across eight European countries. *Diabetologia*. 2018; 61: 1325-32.

25 Meidtner K, Podmore C, Kroger J, van der Schouw YT, Bendinelli B, Agnoli C*, et al.* Interaction of Dietary and Genetic Factors Influencing Body Iron Status and Risk of Type 2 Diabetes Within the EPIC-InterAct Study. *Diabetes Care*. 2018; 41: 277-85.

26 Pasquale LR, Loomis SJ, Aschard H, Kang JH, Cornelis MC, Qi L*, et al.* Exploring genome-wide - dietary heme iron intake interactions and the risk of type 2 diabetes. *Front Genet*. 2013; 4: 7.

27 Qi L, Cornelis MC, Zhang C, van Dam RM, Hu FB. Genetic predisposition, Western dietary pattern, and the risk of type 2 diabetes in men. *American Journal of Clinical Nutrition*. 2009; 89: 1453-58.

28 Qi L, Meigs J, Manson JE, Ma J, Hunter D, Rifai N*, et al.* HFE genetic variability, body iron stores, and the risk of type 2 diabetes in US women. *Diabetes*. 2005; 54: 3567-72.

29 Sonestedt E, Lyssenko V, Ericson U, Gullberg B, Wirfalt E, Groop L*, et al.* Genetic Variation in the Glucose-Dependent Insulinotropic Polypeptide Receptor Modifies the Association between Carbohydrate and Fat Intake and Risk of Type 2 Diabetes in the Malmo Diet and Cancer Cohort. *Journal of Clinical Endocrinology & Metabolism*. 2012; 97: E810-E18.

30 Song Y, Hsu YH, Niu T, Manson JE, Buring JE, Liu S. Common genetic variants of the ion channel transient receptor potential membrane melastatin 6 and 7 (TRPM6 and TRPM7), magnesium intake, and risk of type 2 diabetes in women. *BMC Med Genet*. 2009; 10: 1471-2350.

31 van Hoek M, Dehghan A, Zillikens MC, Hofman A, Witternan JC, Sijbrands EJG. An RBP4 promoter polymorphism increases risk of type 2 diabetes. *Diabetologia*. 2008; 51: 1423-28.

32 Villegas R, Delahanty R, Gao YT, Long J, Williams SM, Xiang YB*, et al.* Joint effect of genetic and lifestyle risk factors on type 2 diabetes risk among Chinese men and women. *PLoS One*. 2012; 7: e49464.

33 Villegas R, Williams S, Gao Y, Cai Q, Li H, Elasy T*, et al.* Peroxisome proliferator-activated receptor delta (PPARD) genetic variation and type 2 diabetes in middle-aged Chinese women. *Ann Hum Genet*. 2011; 75: 621-9.

34 Villegas R, Williams SM, Gao Y-T, Long J, Shi J, Cai H*, et al.* Genetic Variation in the Peroxisome Proliferator-Activated Receptor (PPAR) and Peroxisome Proliferator-Activated Receptor Gamma Co-activator 1 (PGC1) Gene Families and Type 2 Diabetes. *Annals of Human Genetics*. 2014; 78: 23-32.

35 Wirstrom T, Hilding A, Gu HF, Ostenson C-G, Bjorklund A. Consumption of whole grain reduces risk of deteriorating glucose tolerance, including progression to prediabetes. *American Journal of Clinical Nutrition*. 2013; 97: 179-87.

36 Billings LK, Jablonski KA, Ackerman RJ, Taylor A, Fanelli RR, McAteer JB*, et al.* The Influence of Rare Genetic Variation in SLC30A8 on Diabetes Incidence and beta-Cell Function. *Journal of Clinical Endocrinology & Metabolism*. 2014; 99: E926-E30.

37 Bo S, Gambino R, Ciccone G, Rosato R, Milanesio N, Villois P*, et al.* Effects of TCF7L2 polymorphisms on glucose values after a lifestyle intervention. *American Journal of Clinical Nutrition*. 2009; 90: 1502-08.

38 Corella D, Asensio EM, Coltell O, Sorli JV, Estruch R, Martinez-Gonzalez MA*, et al.* CLOCK gene variation is associated with incidence of type-2 diabetes and cardiovascular diseases in type-2 diabetic subjects: dietary modulation in the PREDIMED randomized trial. *Cardiovasc Diabetol*. 2016; 15: 4.

39 de Mello VDF, Lindstrom J, Eriksson JG, Ilanne-Parikka P, Keinanen-Kiukaanniemi S, Pihlajamaki J*, et al.* Markers of cholesterol metabolism as biomarkers in predicting diabetes in the Finnish Diabetes Prevention Study. *Nutrition, Metabolism and Cardiovascular Diseases*. 2015; 25: 635-42.

40 Florez JC, Jablonski KA, Bayley N, Pollin TI, de Bakker PIW, Shuldiner AR*, et al.* TCF7L2 polymorphisms and progression to diabetes in the Diabetes Prevention Program. *New England Journal of Medicine*. 2006; 355: 241-50.

41 Florez JC, Jablonski KA, Sun MW, Bayley N, Kahn SE, Shamoon H*, et al.* Effects of the type 2 diabetes-associated PPARG P12A polymorphism on progression to diabetes and response to troglitazone. *Journal of Clinical Endocrinology & Metabolism*. 2007; 92: 1502-09.

42 Florez JC, Jablonski KA, McAteer J, Sandhu MS, Wareham NJ, Barroso I*, et al.* Testing of diabetes-associated WFS1 polymorphisms in the Diabetes Prevention Program. *Diabetologia*. 2008; 51: 451-57.

43 Florez JC, Jablonski KA, McAteer JB, Franks PW, Mason CC, Mather K*, et al.* Effects of genetic variants previously associated with fasting glucose and insulin in the Diabetes Prevention Program. *PLoS One*. 2012; 7: e44424.

44 Florez JC, Jablonski KA, Kahn SE, Franks PW, Dabelea D, Hamman RF*, et al.* Type 2 diabetes-associated missense polymorphisms KCNJ11 E23K and ABCC8 A1369S influence progression to diabetes and response to interventions in the diabetes prevention program. *Diabetes*. 2007; 56: 531-36.

45 Florez JC, Jablonski KA, Taylor A, Mather K, Horton E, White NH*, et al.* The C Allele of ATM rs11212617 Does Not Associate With Metformin Response in the Diabetes Prevention Program. *Diabetes Care*. 2012; 35: 1864-67.

46 Hivert M-F, Jablonski KA, Perreault L, Saxena R, McAteer JB, Franks PW*, et al.* Updated Genetic Score Based on 34 Confirmed Type 2 Diabetes Loci Is Associated With Diabetes Incidence and Regression to Normoglycemia in the Diabetes Prevention Program. *Diabetes*. 2011; 60: 1340-48.

47 Jablonski KA, McAteer JB, de Bakker PIW, Franks PW, Pollin TI, Hanson RL*, et al.* Common Variants in 40 Genes Assessed for Diabetes Incidence and Response to Metformin and Lifestyle Intervention in the Diabetes Prevention Program. *Diabetes*. 2010; 59: 2672-81.

48 Kilpelaeinen TO, Lakka TA, Laaksonen DE, Laukkanen O, Lindstroem J, Eriksson JG*, et al.* Physical activity modifies the effect of SNPs in the SLC2A2 (GLUT2) and ABCC8 (SUR1) genes on the risk of developing type 2 diabetes. *Physiological Genomics*. 2007; 31: 264-72.

49 Kilpelaeinen TO, Lakka TA, Laaksonen DE, Lindstrom J, Eriksson JG, Valle TT*, et al.* SNPs in PPARG associate with type 2 diabetes and interact with physical activity. *Medicine and science in sports and exercise*. 2008; 40: 25-33.

50 Kilpelaeinen TO, Lakka TA, Laaksonen DE, Mager U, Salopuro T, Kubaszek A*, et al.* Interaction of single nucleotide polymorphisms in ADRB2, ADRB3, TNF, IL6, IGF1R, LIPC, LEPR, and GHRL with physical activity on the risk of type 2 diabetes mellitus and changes in characteristics of the metabolic syndrome: The Finnish Diabetes Prevention Study. *Metabolism-Clinical and Experimental*. 2008; 57: 428-36.

51 Kubaszek A, Pihlajamaki J, Komarovski V, Lindi V, Lindstrom J, Eriksson J*, et al.* Promoter polymorphisms of the TNF-alpha (G-308A) and IL-6 (C-174G) genes predict the conversion from impaired glucose tolerance to type 2 diabetes - The Finnish Diabetes Prevention Study. *Diabetes*. 2003; 52: 1872-76.

52 Laaksonen DE, Siitonen N, Lindstrom J, Eriksson JG, Reunanen P, Tuomilehto J*, et al.* Physical activity, diet, and incident diabetes in relation to an ADRA2B polymorphism. *Medicine and science in sports and exercise*. 2007; 39: 227-32.

53 Lappalainen TJ, Tolppanen AM, Kolehmainen M, Schwab U, Lindstrom J, Tuomilehto J*, et al.* The common variant in the FTO gene did not modify the effect of lifestyle changes on body weight: the Finnish Diabetes Prevention Study. *Obesity (Silver Spring)*. 2009; 17: 832-6.

54 Mather KJ, Christophi CA, Jablonski KA, Knowler WC, Goldberg RB, Kahn SE*, et al.* Common variants in genes encoding adiponectin (ADIPOQ) and its receptors (ADIPOR1/2), adiponectin concentrations, and diabetes incidence in the Diabetes Prevention Program. *Diabet Med*. 2012; 29: 1579-88.

55 Moore AF, Jablonski KA, Mason CC, McAteer JB, Arakaki RF, Goldstein BJ*, et al.* The Association of ENPP1 K121Q with Diabetes Incidence Is Abolished by Lifestyle Modification in the Diabetes Prevention Program. *Journal of Clinical Endocrinology & Metabolism*. 2009; 94: 449-55.

56 Moore AF, Jablonski KA, McAteer JB, Saxena R, Pollin TI, Franks PW*, et al.* Extension of type 2 diabetes genome-wide association scan results in the Diabetes Prevention Program. *Diabetes*. 2008; 57: 2503-10.

57 Pan Q, Delahanty LM, Jablonski KA, Knowler WC, Kahn SE, Florez JC*, et al.* Variation at the melanocortin 4 receptor gene and response to weight-loss interventions in the diabetes prevention program. *Obesity (Silver Spring)*. 2013; 21: E520-6.

58 Pollin TI, Jablonski KA, McAteer JB, Saxena R, Kathiresan S, Kahn SE*, et al.* Triglyceride Response to an Intensive Lifestyle Intervention Is Enhanced in Carriers of the GCKR Pro446Leu Polymorphism. *Journal of Clinical Endocrinology & Metabolism*. 2011; 96: E1142-E47.

59 Salopuro T, Lindstrom J, Eriksson JG, Valle TT, Hamalainen H, Ilanne-Parikka P*, et al.* Common variants in beta2- and beta3-adrenergic receptor genes and uncoupling protein 1 as predictors of the risk for type 2 diabetes and body weight changes. The Finnish Diabetes Prevention Study. *Clin Genet*. 2004; 66: 365-7.

60 Salopuro T, Pulkkinen L, Lindstrom J, Eriksson JG, Valle TT, Hamalainen H*, et al.* Genetic variation in leptin receptor gene is associated with type 2 diabetes and body weight: The Finnish Diabetes Prevention Study. *Int J Obes (Lond)*. 2005; 29: 1245-51.

61 Siitonen N, Lindstrom J, Eriksson J, Valle TT, Hamalainen H, Ilanne-Parikka P*, et al.* Association between a deletion/insertion polymorphism in the alpha 2B-adrenergic receptor gene and insulin secretion and Type 2 diabetes. The Finnish Diabetes Prevention Study. *Diabetologia*. 2004; 47: 1416-24.

62 Siitonen N, Pulkkinen L, Lindstrom J, Kolehmainen M, Eriksson JG, Venojarvi M*, et al.* Association of ADIPOQ gene variants with body weight, type 2 diabetes and serum adiponectin concentrations: the Finnish Diabetes Prevention Study. *Bmc Medical Genetics*. 2011; 12.

63 Siitonen N, Pulkkinen L, Lindstrom J, Kolehmainen M, Schwab U, Eriksson JG*, et al.* Association of ADIPOR2 gene variants with cardiovascular disease and type 2 diabetes risk in individuals with impaired glucose tolerance: the Finnish Diabetes Prevention Study. *Cardiovascular Diabetology*. 2011; 10.

64 Todorova B, Kubaszek A, Pihlajamaki J, Lindstrom J, Eriksson J, Valle TT*, et al.* The G-250A promoter polymorphism of the hepatic lipase gene predicts the conversion from impaired glucose tolerance to type 2 diabetes mellitus: The Finnish Diabetes Prevention Study. *Journal of Clinical Endocrinology & Metabolism*. 2004; 89: 2019-23.

65 Uusitupa MI, Stancakova A, Peltonen M, Eriksson JG, Lindstrom J, Aunola S*, et al.* Impact of positive family history and genetic risk variants on the incidence of diabetes: The Finnish diabetes prevention study. *Diabetes Care*. 2011; 34: 418-23.

66 Wang J, Kuusisto J, Vanttinen M, Kuulasmaa T, Lindstrom J, Tuomilehto J*, et al.* Variants of transcription factor 7-like 2 (TCF7L2) gene predict conversion to type 2 diabetes in the Finnish Diabetes Prevention Study and are associated with impaired glucose regulation and impaired insulin secretion. *Diabetologia*. 2007; 50: 1192-200.

67 Chan KHK, Chacko SA, Song Y, Cho M, Eaton CB, Wu W-CH*, et al.* Genetic Variations in Magnesium-Related Ion Channels May Affect Diabetes Risk among African American and Hispanic American Women. *Journal of Nutrition*. 2015; 145: 418-24.

68 Lindi VI, Uusitupa MI, Lindstrom J, Louheranta A, Eriksson JG, Valle TT*, et al.* Association of the Pro12Ala polymorphism in the PPAR-gamma2 gene with 3-year incidence of type 2 diabetes and body weight change in the Finnish Diabetes Prevention Study. *Diabetes*. 2002; 51: 2581-6.

69 Laukkanen O, Pihlajamaki J, Lindstrom J, Eriksson J, Valle TT, Hamalainen H*, et al.* Polymorphisms of the SUR1 (ABCC8) and Kir6.2 (KCNJ11) genes predict the conversion from impaired glucose tolerance to type 2 diabetes. The Finnish Diabetes Prevention Study. *J Clin Endocrinol Metab*. 2004; 89: 6286-90.

70 Laukkanen O, Pihlajamaki J, Lindstrom J, Eriksson J, Valle TT, Hamalainen H*, et al.* Common polymorphisms in the genes regulating the early insulin signalling pathway: effects on weight change and the conversion from impaired glucose tolerance to Type 2 diabetes. The Finnish Diabetes Prevention Study. *Diabetologia*. 2004; 47: 871-77.

71 Laukkanen O, Lindstrom J, Eriksson J, Valle TT, Hamalainen H, Ilanne-Parikka P*, et al.* Polymorphisms in the SLC2A2 (GLUT2) gene are associated with the conversion from impaired glucose tolerance to type 2 diabetes: the Finnish Diabetes Prevention Study. *Diabetes*. 2005; 54: 2256-60.

72 Mager U, Lindi V, Lindstrom J, Eriksson JG, Valle TT, Hamalainen H*, et al.* Association of the Leu72Met polymorphism of the ghrelin gene with the risk of Type 2 diabetes in subjects with impaired glucose tolerance in the Finnish Diabetes Prevention Study. *Diabet Med*. 2006; 23: 685-9.
